# Semaphorin3A Exacerbates Cardiac Microvascular Rarefaction in Pressure Overload‐Induced Heart Disease

**DOI:** 10.1002/advs.202206801

**Published:** 2023-06-13

**Authors:** Chaofu Li, Yongchao Zhao, Fuhai Li, Zimu Wang, Zhimei Qiu, Yukun Yang, Weidong Xiong, Rui Wang, Han Chen, Fei Xu, Tongtong Zang, Zhiqiang Pei, Yan Wang, Bei Shi, Li Shen, Junbo Ge

**Affiliations:** ^1^ Department of Cardiology Zhongshan Hospital Fudan University Shanghai Institute of Cardiovascular Diseases 180 Fenglin Road, Xuhui District Shanghai 20032 P. R. China; ^2^ Department of Cardiology Affiliated Hospital of Qingdao University Qingdao 266000 P. R. China; ^3^ Department of Cardiology Affiliated Hospital of Zunyi Medical University Zunyi 563000 P. R. China; ^4^ The neuroscience lab University Hospital Essen University of Duisburg‐Essen D‐45122 Essen Germany

**Keywords:** microvascular endothelial cells, microvascular rarefaction, overload‐induced heart disease, semaphorin3A, small extracellular vehicles

## Abstract

Microvascular endothelial cells (MiVECs) impair angiogenic potential, leading to microvascular rarefaction, which is a characteristic feature of chronic pressure overload‐induced cardiac dysfunction. Semaphorin3A (Sema3A) is a secreted protein upregulated in MiVECs following angiotensin II (Ang II) activation and pressure overload stimuli. However, its role and mechanism in microvascular rarefaction remain elusive. The function and mechanism of action of Sema3A in pressure overload‐induced microvascular rarefaction, is explored, through an Ang II‐induced animal model of pressure overload. RNA sequencing, immunoblotting analysis, enzyme‐linked immunosorbent assay, quantitative reverse transcription polymerase chain reaction (qRT‐PCR), and immunofluorescence staining results indicate that Sema3A is predominantly expressed and significantly upregulated in MiVECs under pressure overload. Immunoelectron microscopy and nano‐flow cytometry analyses indicate small extracellular vesicles (sEVs), with surface‐attached Sema3A, to be a novel tool for efficient release and delivery of Sema3A from the MiVECs to extracellular microenvironment. To investigate pressure overload‐mediated cardiac microvascular rarefaction and cardiac fibrosis in vivo, endothelial‐specific *Sema3A* knockdown mice are established. Mechanistically, serum response factor (transcription factor) promotes the production of Sema3A; Sema3A‐positive sEVs compete with vascular endothelial growth factor A to bind to neuropilin‐1. Therefore, MiVECs lose their ability to respond to angiogenesis. In conclusion, Sema3A is a key pathogenic mediator that impairs the angiogenic potential of MiVECs, which leads to cardiac microvascular rarefaction in pressure overload‐induced heart disease.

## Graphical abstract

The schematic presentation depicts the mechanism by which the Semaphorin3A (Sema3A)‐positive small extracellular vesicle (sEV)/neuropilin‐1 (NRP1) axis meditates microvascular rarefaction. The mechanism of impaired angiogenesis involves Sema3A‐positive sEVs competing with vascular endothelial growth factor A for binding to NRP1, to inhibit the adaptive angiogenic response. In addition, communication between microvascular endothelial cells and fibroblasts via Sema3A‐containing sEVs causes myofibroblast activation and promotes the development and progression of cardiac fibrosis.

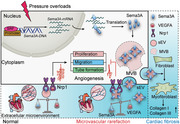

## 1 Introduction

**Scheme 1:**
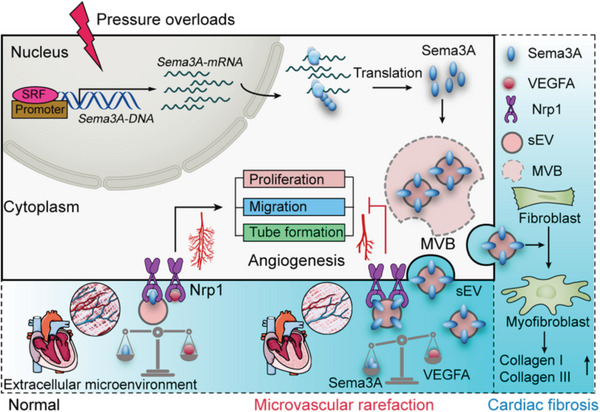
Schematic diagram of this study This schematic presentation depicts the mechanism by which the Sema3A‐positive sEV/NRP1 axis meditates microvascular rarefaction. The mechanism of impaired angiogenesis involves Sema3A‐positive sEVs competing with VEGF‐A to bind to NRP1, thus inhibiting the adaptive angiogenic response. In addition, communication between MiVECs and fibroblasts via Sema3A‐containing sEVs causes myofibroblast activation and promotes the development and progression of cardiac fibrosis.

Given the increasing incidence of aging and hypertension, sustained chronic pressure overload that triggers cardiac remodeling has become a global public health issue.^[^ ^1^, ^2^ ^]^ Cardiac hypertrophy, interstitial fibrosis, and microvascular rarefaction are the key pathological processes of cardiac remodeling induced by chronic pressure overload.^[^ ^3^, ^4^ ^]^ Previously, an established view was that the “dilutive effect” of increased cardiomyocyte size on microvessels leads to reduced vessel density.^[^ ^4^, ^5^ ^]^ Many studies have focused on ameliorating cardiac hypertrophy by reversing cardiomyocyte size.^[^ ^6^, ^7^, ^8^ ^]^ However, state‐of‐the‐art therapies failing to reverse hypertrophic remodeling emphasizes the need for better understanding of hypertrophic cardiac remodeling. **Scheme** **1**

Recently, accumulating evidence has suggested the involvement of maladaptive angiogenic response in microvascular rarefaction post pressure overload.^[^ ^9^, ^10^ ^]^ In other words, microvascular endothelial cells (MiVECs) with impaired angiogenic potential fail to create new blood vessels to maintain adequate oxygen and nutrients to sustain hypertrophic myocardial tissue.^[^ ^11^ ^]^ However, the underlying mechanism of this impaired angiogenic potential in microvascular rarefaction remain poorly understood.

Multivesicular bodies (MVBs) are key for the biogenesis of small extracellular vehicles (sEVs) and the release of sEVs into the extracellular space.^[^ ^12^, ^13^ ^]^ sEVs are endosome originated vesicles that transfer lipids, nucleic acids, and proteins between different cells, tissues, or organs, which are crucial for intercellular communication.^[^ ^14^ ^]^ Our previous study also found that sEVs packaged with miR‐29a could derived from myocytes in hypertrophic hearts, and inhibit the angiogenic potential of MiVECs.^[^ ^15^ ^]^ Moreover, it is worth noting that sEVs can act as cargo carriers and are involved in protein secretion. However, it is unclear whether sEVs participate in cardiac remodeling. Semaphorin3A (Sema3A), a protein belonging to the semaphorin family, can be secreted into extracellular environment and anchored to corresponding membrane receptor.^[^ ^16^, ^17^, ^18^ ^]^ The role and underlying mechanisms of Sema3A in cardiac remodeling has not been investigated. Furthermore, whether Sema3A are imported into vesicle and exported to extracellular environment remain poorly understood.

In the present study, we investigated the role and related mechanisms of Sema3A in microvascular rarefaction under pressure overload conditions. Both in vitro and in vivo, Sema3A depletion reduced pressure overload‐induced microvascular rarefaction. Furthermore, immunoelectron microscopy revealed that Sema3A was located in MVB‐like vesicles. Additionally, Sema3A was found to be expressed at the surface of MiVEC‐derived sEVs and was upregulated following angiotensin II (Ang II) stimuli. Notably, sEVs have been proven to be a novel platform for the delivery and release of Sema3A from MiVECs to extracellular environment, thus mediating capillary rarefaction. Collectively, these findings suggest that Sema3A‐positive sEVs play a crucial role in driving endothelial cell injury and vascular rarefaction during pressure overload‐induced cardiac remodeling. Moreover, our study findings conceptually advance the understanding of pathophysiology of microvascular rarefaction and provide a candidate translational option for strategies to mitigate capillary loss in pressure overload‐induced heart disease.

## 2 Results

### 2.1 Angiogenic Capacity and Transcriptome Alterations of Microvascular Endothelial Cells in Cardiac Hypertrophy

**Figure 1:**
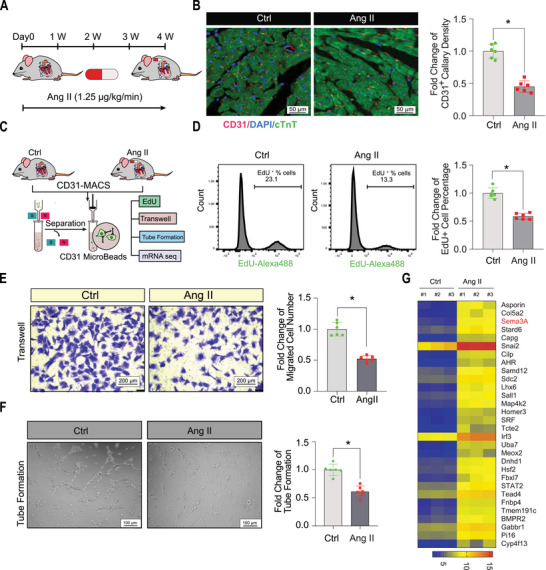
Angiogenic capacity and transcriptome alterations of MiVECs in cardiac hypertrophy. A) Schematic diagram of angiotensin II (Ang II)‐induced cardiac diastolic dysfunction in mice and its detection at the corresponding time points. B) Capillaries were stained with the endothelial cell marker CD31 (red), myocardial tissue was stained with cTnt (green), and nuclei were counterstained with DAPI (blue). Scale bar = 50 µm. *n* = 6 mice per group. C) Overview of the experimental design for isolation and detection of MiVECs in vitro. D) MiVEC proliferation evaluation performed using the EdU assay. *n* = 6 mice per group. E) Representative images of migrated MiVECs by the transwell assay. Scale bar = 200 µm. *n* = 6 mice per group. F) Representative images and quantification of MiVECs in the tube formation experiment, Scale bar = 100 µm. *n* = 6 mice per group. G) Heatmap of RNA‐sequencing analysis showed that gene expression was significantly upregulated in MiVECs isolated from the hearts of the Ang II‐induced group compared with the saline control group. For all statistical plots, the data are presented as the mean ± SEM. *p < 0.05 between the two indicated groups by 2‐tailed Student's *t*‐test. cTnT, cardiac troponin T; EdU, 5‐ethynyl‐2′‐deoxyuridine; DAPI, 4′,6‐diamidino‐2‐phenylindole; MACS, magnetic‐activated cell sorting.

We established a pressure overload mouse model infused with Ang II to characterize angiogenesis‐related angiogenesis alterations in cardiac hypertrophy and induced cardiac hypertrophy and capillary rarefaction. Adult mice received infusions of Ang II or saline control, over a period of 4 weeks, delivered via a subcutaneous osmotic mini‐pump (**Figure** **1**A). The mice developed progressive cardiac hypertrophy with enlarged heart size (Figure S1A, Supporting Information), weight (Figure S1B,C, Supporting Information), cell surface area (Figure S1D, Supporting Information), and marked capillary rarefaction (Figure 1B) after 4 weeks of Ang II infusion. We isolated and characterized mouse cardiac MiVECs from Ang II‐treated and saline control mice (Figure S2A–E, Supporting Information). Next, EdU incorporation, transwell migration, and Matrigel tube formation assays were performed to evaluate their proliferation, migration, and tubule formation, respectively (Figure 1C). The results showed that the proliferation (Figure 1D), migration (Figure 1E), and tubulogenesis (Figure 1F) of myocardial MiVECs in the Ang II‐induced group were significantly reduced compared to those in the saline control group. Further, we observed gene expression changes in MiVECs in capillary rarefaction hearts. RNA sequencing of the isolated MiVECs revealed a greater than twofold upregulation of expression of 30 genes (*p* < 0.01 versus the saline control). The most abundantly upregulated genes in terms of fold‐change and significance were *AsporinA*, *Col5a2*, *Sema3a*, *Stard6*, and *Capg* (Figure 1G). Overall, these results suggest that angiogenic dysfunction in MiVECs may play a critical role in regulating cardiac hypertrophy and capillary rarefaction.

### 2.2 Kinetics of Semaphorin3A Expression in Cardiac Pressure Overload‐Induced Cardiac Hypertrophy

**Figure 2:**
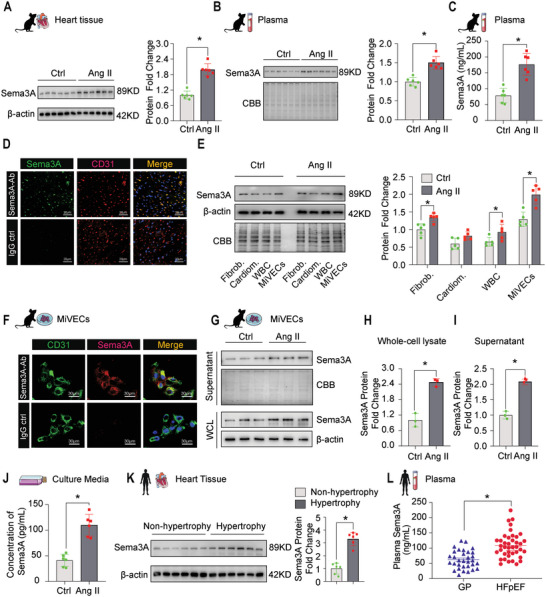
Kinetics of Sema3A expression in cardiac pressure overload‐induced cardiac hypertrophy. A) Representative images of immunoblotting analysis of Sema3A protein expression in Ang II‐infused mouse hearts after 4 weeks. The protein expression was quantified and normalized to that of *β*‐actin. *n* = 6 mice per group. B) Representative immunoblotting of Sema3A levels after 4 weeks in serum collected by cardiac puncture from an Ang II‐infused mouse heart. Blots stained with Coomassie brilliant blue served as loading controls (*n* = 6 mice per group). C) Sema3A serum concentration (pg mL^−1^) measured using ELISA; *n* = 6 mice per group. D) Double immunofluorescence staining of Sema3A (green) and CD31 (red) in Ang II‐infused mouse hearts. The nuclei were stained with DAPI (blue). Immunoglobulin G (IgG) antibodies were used as negative controls. Scale bar = 20 µm. E) Immunoblotting analysis of the expression of Sema3A in cardiac fibroblasts (Fibrob.), cardiomyocytes (Cardiom.), white blood cells (WBC), and MiVECs isolated directly from the heart. *n* = 5 mice per group. F) Representative double immunofluorescence staining images of Sema3A (red) and the vascular endothelial cell marker CD31 (green) in MiVECs. The nuclei were stained with DAPI (blue). Immunoglobulin G (IgG) antibodies were used as negative controls. Scale bar = 30 µm. G) Representative immunoblotting images and H) quantification of Sema3A protein expression in MiVECs and I) the culture medium with or without Ang II treatment (1 µM for 24 h); *n* = 3 samples per group. J) Sema3A protein secreted into the cell culture medium, as measured by ELISA; *n* = 3 samples per group. K) Immunoblotting analysis of Sema3A expression in human samples from non‐hypertrophic and hypertrophic myocardial tissues (*n* = 6). L) Circulating Sema3A levels in the general population (*n* = 32) and in patients with heart failure with preserved ejection fraction (*n* = 40). Each data point represents a serum sample, the horizontal middle line in each data set represents the median, and the limits of the vertical lines represent the interquartile range. For all statistical plots, the data are presented as the mean ± SEM; *p < 0.05, between the two indicated groups by 2‐tailed Student's *t*‐test. CBB, Coomassie brilliant blue staining; IgG, immunoglobulin G; GP, General population; HFpEF, heart failure with preserved ejection fraction.

RNA sequencing analysis revealed that Sema3A is a highly upregulated gene in MiVECs after Ang II infusion. Sema3A, a member of a larger family comprising of six secreted glycoproteins, is located in the cell membrane and can be secreted into the extracellular milieu. To explore the expression profile of cardiac Sema3A during pressure overload, we examined Sema3A content at various time points (1 day, 3 days, 1 week, 2 weeks, 4 weeks, and 6 weeks) in Ang II‐infused mouse hearts. Sema3A levels increased from the first week, peaked in the fourth week, but considerably decreased after six weeks (Figure S3A, Supporting Information). Given that Sema3A is a secreted protein, we also examined its levels in the serum. The data showed that Sema3A was markedly elevated in both cardiac tissue (**Figure** **2**A) and plasma (Figure 2B,C) after 4 weeks of Ang II infusion. To confirm whether this increase was specific to MiVECs, immunofluorescence staining was performed. The findings revealed that Sema3A was mainly co‐localized with the endothelial cell marker CD31 in Ang II‐infused hearts (Figure 2D). Moreover, we isolated and identified four major cardiac cell types, namely, cardiac fibroblasts, cardiomyocytes, white blood cells, and MiVECs, from the saline control and Ang II‐infused mouse hearts (Figure S2B–K, Supporting Information). Immunoblotting (Figure 2E) and quantitative real‐time polymerase chain reaction (Figure S3B, Supporting Information) demonstrated that MiVECs had a strong ability to upregulate Sema3A expression after infusion with Ang II. MiVECs were treated with Ang II in vitro to mimic in vivo responses. Consistent with the in vivo data, the CCK‐8 assay revealed that MiVEC viability decreased significantly after 24 h of Ang II treatment (1 µM) (Figure S3C, Supporting Information). Immunofluorescence images showed that Sema3A was localized in the cytoplasm and membrane of MiVECs (Figure 2F). Sema3A protein expression was significantly increased in both Ang II‐treated MiVECs (Figure 2G,H) and the culture medium (Figure 2I,J) compared to that in the phosphate‐buffer saline (PBS) control. To test the translational potential of our findings, we analyzed Sema3A expression in the heart and serum of transverse aortic constriction (TAC) mice. Immunoblotting and enzyme‐linked immunosorbent assay (ELISA) analyses revealed that Sema3A expression was upregulated in the heart tissue and serum of mice 4 weeks after cardiac hypertrophy was induced through TAC (Figure S3E–G, Supporting Information). We also analyzed Sema3A expression in human and spontaneously hypertensive rat (SHR) samples. Consistent with these mouse data, Sema3A expression was significantly higher in the hypertrophic hearts (Figure 2K and Figure S3I, Supporting Information) and plasma (Figure S3J, Supporting Information) of patients, in the serum of patients with hypertension‐related cardiac dysfunction (Figure S3L,M, Supporting Information), and in the hearts of SHRs (Figure S3H, Supporting Information) compared to the corresponding controls. Sema3A expression was upregulated in the plasma of patients with heart failure with preserved ejection fraction (Figure 2L), and Sema3A concentrations were positively correlated with NT‐proBNP in these patients (Figure S3K, Supporting Information). Overall, these findings demonstrated that Sema3A levels in MiVECs are elevated during pressure overload‐induced myocardial hypertrophy and that this secretory protein may be involved in microvascular rarefaction.

### 2.3 Depletion of Semaphorin3A Attenuated the Angiotensin II‐Induced Dysfunction of Microvascular Endothelial Cells

**Figure 3:**
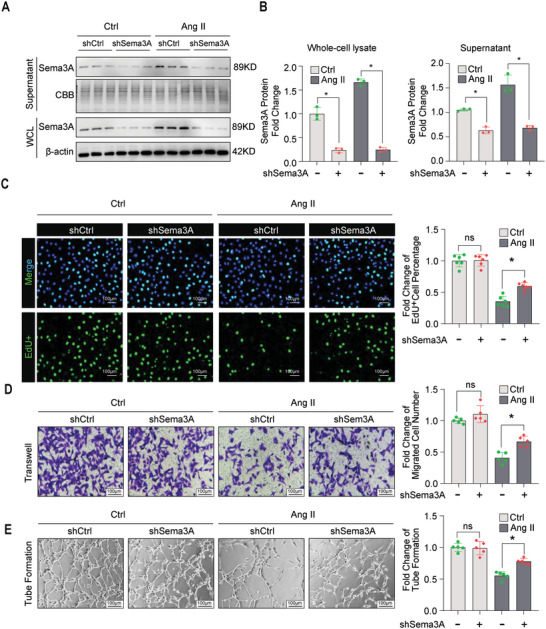
Loss of Sema3A attenuated the Ang II‐Induced dysfunction of MiVECs. A,B) Representative immunoblotting and quantification of Sema3A protein expression after lentivirus carrying Sema3A shRNA (shSema3A) and control shRNA (shCtrl) transfection in the presence or absence of Ang II treatment. *n* = 3 samples per group. C) Representative images of EdU incorporation assays (left panel) and quantification of EdU‐positive cells (right panel). Scale bar = 100 µm. *n* = 6 samples per group. D) Representative images of transwell migration assays and quantification of the migrated number of MiVECs after Sema3A knockdown. Scale bar = 100 µm. *n* = 5 samples per group. E) Representative images and quantification of MiVECs in the tube formation experiment after Sema3A knockdown. Scale bar = 100 µm. *n* = 5 samples per group. *p < 0.05, versus shCtrl group; *n* = 5. All data are presented as mean ± SEM, *p < 0.05. ns indicates no significant difference between the two indicated groups by one‐way analysis ANOVA. WCL, whole cell lysate. CBB, Coomassie brilliant blue staining; EdU, 5‐ethynyl‐2′‐deoxyuridine.

Sema3A expression is upregulated in MiVECs of the heart tissue in models of pressure overload. We determined whether Ang II‐induced Sema3A is involved in capillary rarefaction. Three pairs of shRNAs were designed to silence Sema3A expression. After infection with a lentivirus containing Sema3A shRNA, immunoblotting analysis confirmed the knockdown efficiency of Sema3A. Among the three shRNAs, sh‐Sema3A #3 exhibited the highest silencing efficiency (Figure S4A, Supporting Information) and was selected for further experiments. In both normal and Ang II conditions, Sema3A protein levels were knocked down by shSema3A in both the culture medium and cultured MiVECs (**Figure** **3**A,B and Figure S4B, Supporting Information).

Next, we assessed the silencing effect of Sema3A against MiVECs. As demonstrated, downregulation of Sema3A expression was associated with an increased EdU‐positive nuclei ratio in MiVECs under Ang II treatment conditions, but not under treatment with PBS (Figure 3C). Moreover, Sema3A knockdown enhanced the migration of MiVECs under Ang II treatment (Figure 3D). Similarly, tube formation assay demonstrated that the number of tube‐like structures increased in Sema3A‐silenced MiVECs treated with Ang II, but not in those treated with PBS (Figure 3E). Collectively, these data suggest that Sema3A knockdown plays a pivotal role in the prevention of Ang II‐induced impairment of capillary rarefaction.

### 2.4 Serum Response Factor Promotes Semaphorin3A Expression in Microvascular Endothelial Cells

**Figure 4:**
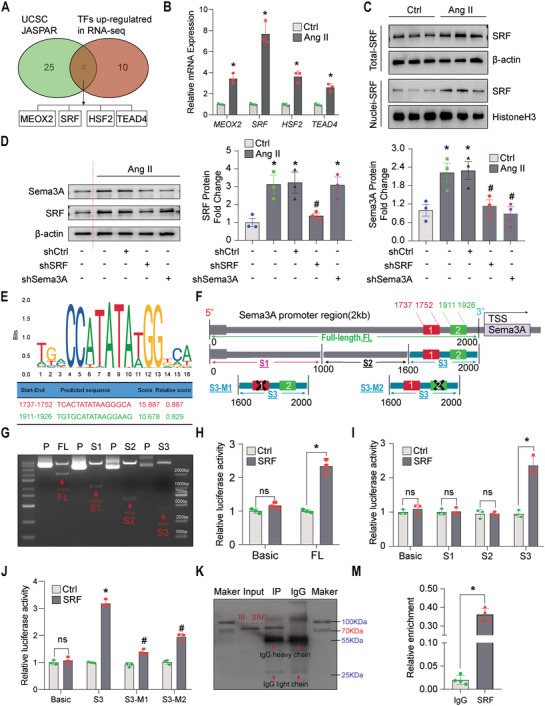
SRF promotes Sema3A expression in MiVECs. A) Venn diagrams of the four transcription factors (TFs) screened from databases and RNA sequencing analysis. B) Quantitative RT‐PCR (RT‐qPCR) analysis of relative mRNA expression (*n* = 3 samples per group). C) Immunoblotting detection of SRF protein expression in total cell lysates and nuclear fractions of MiVECs treated with or without Ang II (1 µM for 24 h). *β*‐actin and Histone H3 were loading controls for the total cell lysate and nuclear extracts, respectively. *p < 0.05, compared to the control group; *n* = 3 samples per group. D) Representative immunoblotting images (left panel) and quantification (right panel) of SRF and Sema3A protein expression after transfection with shSRF or shSema3A after Ang II treatment. *p < 0.05, compared with the control shRNA group; ^#^ *p* < 0.05, versus the control shRNA group; *n* = 3 samples per group. E) SRF binding sequence in the Sema3A promoter region predicted by JASPAR software; the predicted potential binding sequence and score are shown. F) Schematic diagram of the Sema3A promoter and deletion mutants. G) The original plasmid and digested DNA fragments were analyzed by agarose gel electrophoresis. P = plasmid; FL = full‐length plasmid. H) Relative luciferase activity in MiVECs co‐transfected with the luciferase reporter plasmid containing the full‐length promoter sequence and SRF overexpression plasmid. Basic, pGL3 basic plasmid; FL, pGL3‐full‐length promoter; Ctrl, pcDNA3.1‐vector; SRF, pcDNA3.1‐SRF. *p < 0.05, versus pcDNA3.1 control+ pGL3‐full‐length promoter; *n* = 3 samples per group. I,J) Luciferase assay and selective truncation mutation analysis of the Sema3A promoter were used to identify the activation of Sema3A‐containing promoters through SRF. K–M) ChIP‐qPCR analysis of SRF binding to the promoter regions of Sema3A in MiVECs. *n* = 3 samples per group. *p < 0.05. For all statistical plots, the data are presented as mean ± SEM. One‐way analysis of variance (ANOVA) was conducted in (B), (D), (H)–(J); Student's *t*‐test was conducted in (M); ns indicates no significance; IP, immunoprecipitation; IgG, immunoglobulin G.

We investigated the mechanism underlying Sema3A upregulation. Increasing evidence suggests that protein expression is subject to multiple levels of regulation, including protein modification, mRNA stability, and transcription. Given that Sema3A is elevated at both the transcriptional and post‐transcriptional stages, we hypothesized that the elevation of the Sema3A protein might be regulated by transcription factors (TFs). To test this hypothesis, potential TFs were determined using JASPAR (http://jaspar.genereg.net/) and UCSC (http://genome.ucsc.edu/). Next, the candidate TFs were intersected with the TFs from our RNA sequencing data. Eventually, four TFs, namely, MEOX2, serum response factor (SRF), HSF2, and TEAD4, were screened and selected (**Figure** **4**A). Among these candidate TFs, SRF showed the most significant upregulation after Ang II treatment (Figure 4B). Subsequently, knocking down SRF significantly restored the decrease in MiVEC vitality induced by Ang II (Figure S5A, Supporting Information). Additionally, among these candidate TFs, SRF showed the highest efficiency in regulating Sema3A after knockdown (Figure S5B, Supporting Information).

To further explore the relationship between SRF and Sema3A, we examined the protein expression levels of SRF in MiVECs and heart tissue after Ang II treatment. As demonstrated, SRF expression was significantly increased in the Ang II‐treated group in both MiVECs and heart tissue when compared to the respective control group (Figure 4C and Figure S6C, Supporting Information). Nuclear localization is essential for the regulatory functions of TFs. Thus, we isolated nuclei from MiVECs (Figure S6A,B, Supporting Information) and verified the isolation efficiency. Then, the immunoblotting assay verified an increased SRF protein expression in the nucleus of MiVECs under the Ang II treatment (Figure 4C).

To explore the regulatory effects of SRF on Sema3A in MiVECs, lentiviruses carrying SRF shRNA or Sema3A shRNA were used to knock down relative gene expression in MiVECs. An immunoblotting experiment was then performed to detect knockout efficiency (Figure S6D, Supporting Information). ​ The results showed that Sema3A expression was profoundly reduced in MiVECs transfected with SRF shRNA upon Ang II treatment, as compared to the shRNA control, whereas SRF levels showed no significant difference in MiVECs transfected with Sema3A shRNA (Figure 4D). Next, we investigated whether SRF directly interacted with the Sema3A gene promoter. Putative SRF‐binding sites in the Sema3A promoter were predicted through a bioinformatics‐based approach. Based on the predicted sites of SRF in the Sema3A promoter from the JASPAR library (http://jaspar.genereg.net), full‐length (FL), segments truncated (S1–S3), and mutant (MUT) of Sema3A promoter firefly luciferase reporter plasmids were designed and co‐transfected with SRF overexpression plasmids into HEK 293 cells (Figure 4E,F). We found that the SRF protein expression was significantly upregulated after pcDNA3.1‐SRF overexpression plasmid transfection (Figure S6E, Supporting Information). Dual‐luciferase reporter assays showed that SRF promoted the luciferase activity of the full‐length (FL) and S3 segments, but not the S1 and S2 segments of the Sema3A promoter (Figure 4G–J). These results indicated that the promoter region of site1 (S3‐M1) and site2 (S3‐M2) are SRF binding sites. Moreover, SRF could bind to the promoter region of the Sema3A gene and accelerate its transcriptional activity, which was confirmed by ChIP qPCR analysis (Figure 4K–M). Overall, these data support our hypothesis that the upregulation of Sema3A in MiVECs may be transcriptionally induced by SRF.

### 2.5 Serum Response Factor Knockdown Attenuated the Angiotensin II‐Induced Dysfunction of Microvascular Endothelial Cells

**Figure 5:**
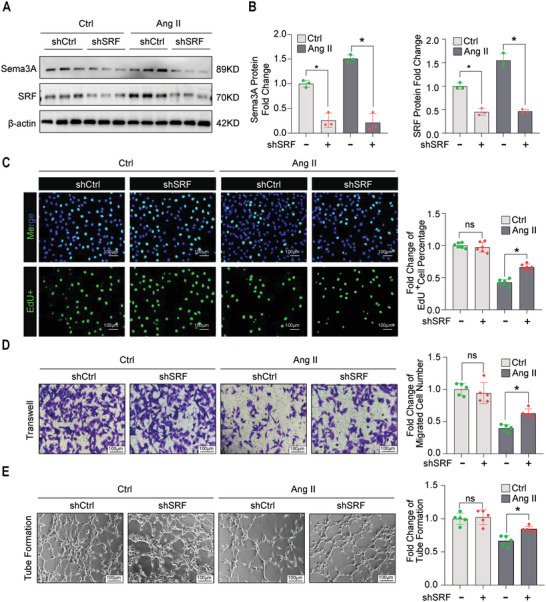
SRF knockdown attenuated the Ang II‐Induced dysfunction of MiVECs. A,B) Representative Immunoblotting showing the knockdown efficiency of SRF shRNA (shSRF) and control shRNA (shCtrl) in with or without Ang II treatment. *n* = 3 samples per group. C) Effect of SRF knockdown tested by shSRF on cell proliferation. Scale bar = 100 µm. *n* = 6 samples per group. D) Representative images of transwell migration assays and quantification of the number of migrated MiVECs after SRF knockdown. Scale bar = 100 µm. *n* = 5 samples per group. E) Representative images and quantification of MiVECs in tube formation experiments after SRF knockdown. Scale bar = 100 µm. *n* = 5 samples per group. All data are presented as mean ± SEM, *p < 0.05. ns indicates no significant difference between the two indicated groups by one‐way analysis ANOVA.

Because SRF can bind to the promoter region of Sema3A and enhance transcription, we explored the potential role of SRF in regulating angiogenesis. Lentivirus‐packaged shRNAs were established and targeting SRF to knock down the levels of SRF in MiVECs with or without Ang II treatment was established (**Figure** **5**A,B). The results showed that SRF knockdown improved MiVEC proliferation and accelerated MiVEC migration under Ang II conditions, as tested by nuclear EdU staining (Figure 5C) and transwell assays (Figure 5D), respectively. Moreover, after SRF knockdown, the number of tube‐like structures in MiVECs increased remarkably (Figure 5E). Collectively, these results implied that SRF inhibited the proliferation, migration, and tube formation of MiVECs in vitro.

### 2.6 Semaphorin3A Impaired Angiogenic Potential by Competing with Vascular Endothelial Growth Factor A to Binding to Neuropilin‐1

**Figure 6:**
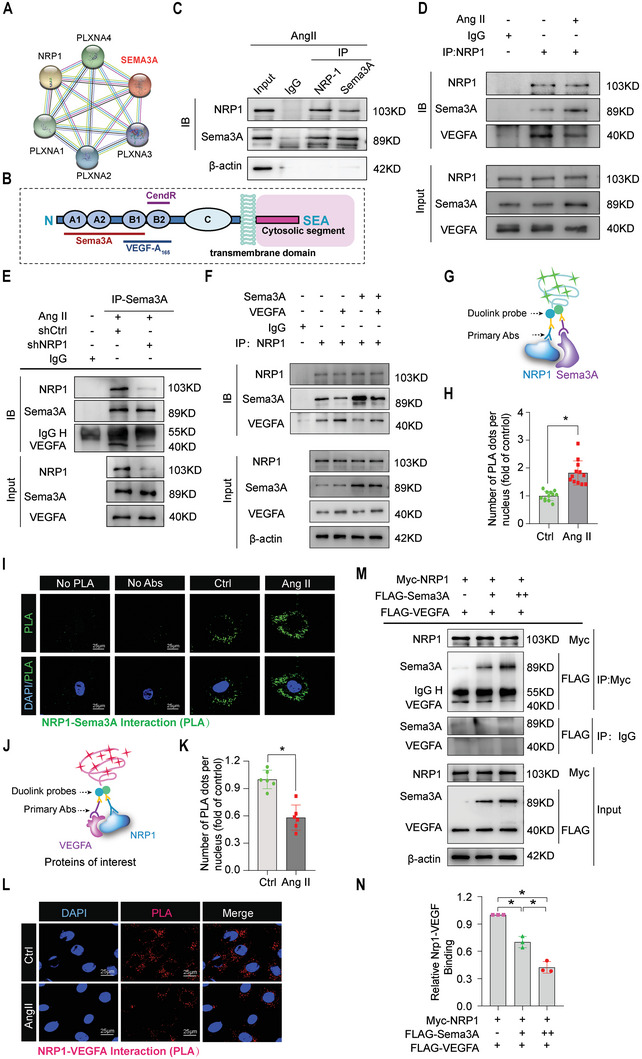
Sema3A impaired angiogenic potential by competitively binding to NRP1 with VEGFA. A) Computational prediction of Sema3A protein partner network visualization on the STRING website. B) General domain structure of NRP1. C) Interaction between endogenous Sema3A and NRP1 detected by co‐immunoprecipitation (co‐IP) experiments in MiVECs treated with Ang II. D) Representative co‐IP analysis of NRP1, Sema3A, and VEGFA in PBS‐treated or Ang II‐treated MiVECs. E) Decreased combination of Sema3A with VEGFA in MiVECs transfected with shNRP1. F) Co‐IP analysis of NRP1, Sema3A, and VEGFA in MiVECs infected with adenovirus (Ad)‐Sema3A or Ad‐VEGFA. G) Schematic representation of proximity ligation assay (PLA) detection and analysis of NRP1 interaction (protein‐protein interaction) with Sema3A in MiVECs treated with or without Ang II. I) Representative images and H) quantification of proximity ligation assay (PLA) analysis showing the interaction of Sema3A with NRP1 in MiVECs. PLA‐positive spots are shown in green. Nuclei were counterstained with DAPI (blue). No antibodies: PLA was performed in the absence of primary antibodies. Scale bar = 25 µm. *n* = 12 samples per group. J) Schematic of PLA detection and analysis of NRP1 interaction with VEGFA. L) Representative images and K) quantification of proximity ligation assay (PLA) detection of endogenous NRP1 and VEGFA interactions in MiVECs. Each red dot represents the detection of the protein‐protein complex, and nuclei were counterstained with DAPI (blue). Scale bar = 25 µm. *n* = 6 samples per group. M) Competitive co‐IP assay and N) quantitative analysis showing significantly reduced NRP1 and VEGFA interaction by overexpression of Sema3A in a dose‐dependent manner in MiVECs. Myc‐NRP1, FLAG‐VEGFA, and FLAG‐Sema3A plasmids were co‐transfected into MiVECs according to the experimental conditions. For all statistical plots, the data are presented as the mean ± SEM, *p < 0.05. ANOVA with Sidak's correction was conducted in (N); Student's *t*‐test was used in (H) and (K). IP indicates immunoprecipitation; IB, Immunoblotting; Abs, antibodies; DAPI, 4′,6‐diamidino‐2‐phenylindole.

We aimed to identify possible downstream targets of Sema3A in MiVECs that might influence the adaptive angiogenic response in the heart after pressure overload. We predicted possible interaction partners of Sema3A according to the STRING database (http://string‐db.org/) (**Figure** **6**A). Among these possible interaction partners, the transmembrane protein Neuropilin‐1 (NRP1) is a receptor shared by Sema3A and vascular endothelial growth factor A (VEGFA) (Figure 6B). Thus, we verified if Sema3A interacted with NRP1 in MiVECs. Co‐immunoprecipitation results showed that endogenous NRP1 combined with Sema3A in MiVECs, after stimulation with Ang II (Figure 6C). Next, we examined the protein levels of SRF and Sema3A in MiVECs and found a significant increase in both following stimulation with Ang II, compared with the PBS controls, whereas no significant difference was observed for NRP1 (Figure S7A, Supporting Information). Considering that Nrp1 is a co‐receptor for Sema3A and VEGFA, we speculated that Sema3A interferes with the association of VEGFA with NRP1 in a competitive manner. Thus, several protein‐protein interaction experiments were performed (Figure 6D–F). We investigated the potential interaction between endogenous proteins in MiVECs, and endogenous immunoprecipitation of NRP1 pulled down Sema3A and VEGFA, indicating that NRP1 interacted with Sema3A and VEGFA (Figure 6D). Concomitant knockdown of endogenous NRP1 in MiVECs (Figure S7B, Supporting Information) led to a decrease in the content of Sema3A‐NRP1‐VEGFA complexes (Figure 6E).

In addition, we found that the amount of endogenous Sema3A‐NRP1 complexes in MiVECs increased significantly after Ang II stimulation compared to the PBS control, VEGFA‐NRP1 expression decreased significantly in Ang II‐stimulated MiVECs (Figure 6D). Consistent with the co‐IP assay results, levels of Sema3A‐NRP1 complexes increased significantly, as confirmed by the proximity ligation assay (Figure 6G–I). Conversely, the levels of VEGFA‐NRP1 complexes decreased in MiVECs upon Ang II treatment, as compared with the PBS‐treated control (Figure 6J–L). In addition, a competitive co‐IP assay in MiVECs showed that Sema3A and VEGFA compete to binding to NRP1 (Figure 6M,N). Overall, these results provide compelling evidence that Sema3A competes with VEGFA to bind to NRP1 and inhibit adaptive angiogenesis in response to pressure overload.

### 2.7 Small Extracellular Vesicles Secreted from Microvascular Endothelial Cells Carrying Semaphorin3A Impaired Angiogenic Potential

**Figure 7:**
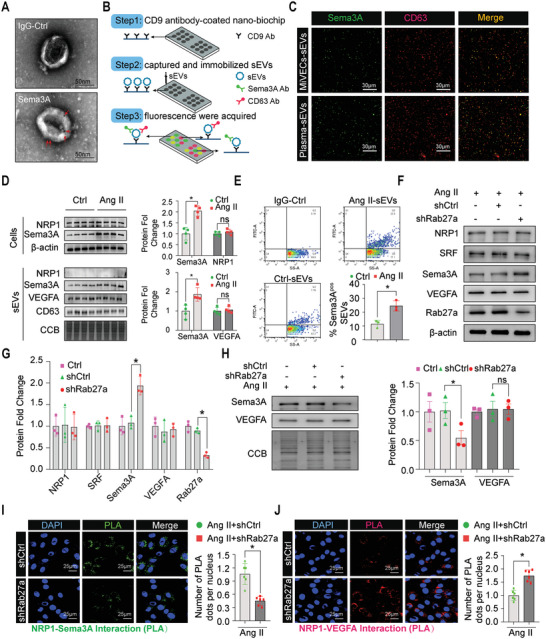
sEV‐mediated secretion of Sema3A impaired angiogenic potential. A) Electronic microscopy analysis of sEVs stained by immunogold for Sema3A. The high‐density dots indicated by the red triangles represent the immunogold labelling of the Sema3A protein. Scale bar = 50 nm. B) Schematic workflow of sEV immunofluorescence staining. C) Immunofluorescence of sEVs from cell supernatants of MiVECs and mouse plasma samples stained with CD63‐Alexa Fluor 647 and Sema3A‐Alexa Fluor 488 antibodies. The yellow spots represent sEVs that contain both CD63 and Sema3A proteins. Scale bar = 30 µm; Ab indicates the antibody. D) Immunoblotting (left panel) and densitometric analysis (right panel) of Sema3A, NRP1, and VEGFA in sEVs obtained from MiVECs with or without treatment with Ang II. *p < 0.05, compared to the control group. *n* = 4 per group. E) Bivariate dot plots of FITC fluorescence versus SS‐A for sEVs preparations from MiVECs. sEVs were fluorescently labelled with FITC‐conjugated mAbs specific to Sema3A. F,G) Rab27a, a key gene involved in extracellular vesicle biogenesis, was silenced by shRNA to block sEVs secretion. F) Immunoblotting images and G) quantification of NRP1, SRF, Sema3A, VEGFA, and Rab27 proteins from MiVECs transfected with control shRNA (shCtrl) or shRab27a upon treatment with Ang II. H) Immunoblotting analysis of Sema3A and VEGFA protein expression changes in the supernatant of MiVECs after blocking sEVS secretion by Rab27a knockdown. Coomassie brilliant blue (CBB) served as the loading control. *n* = 3 per group. I) Proximity ligation assay using antibodies against NRP1 and Sema3A in MiVECs transfected with shCtrl or shRab27a after Ang II treatment. Representative confocal microscopy images (left panel) and quantitative data (right panel) are shown. PLA‐positive spots are shown in green, and the cellular nuclei were counterstained with DAPI (blue). Scale bar = 25 µm. *n* = 6 per group. J) Representative images (upper panel) and quantitative analysis (lower panel) of the proximity ligation assay for NRP1 and VEGFA interactions. PLA‐positive spots are shown in red; Scale bar = 25 µm; *n* = 6 per group. For all statistical plots, the data are presented as mean ± SEM, One‐way ANOVA was conducted in (D), (G), and (H); Student's *t*‐test were conducted in (E), (I), and (J). IgG indicates immunoglobulin G; SS‐A, side scatter‐axis; mAbs, monoclonal antibodies.

Given that Sema3A is a secreted protein, we investigated the gain insight into the underlying mechanism of Sema3A protein secretion. We examined the subcellular localization of Sema3A in MiVECs by immunoelectron microscopy with colloidal gold (Figure S8A,B, Supporting Information). Many Sema3A proteins are localized to MVB‐like vesicles (Figure S8A, Supporting Information), and vesicles that appear ready to undergo exocytosis (Figure S8B, Supporting Information). Additionally, a significant amount of Sema3A was found in the Golgi/trans‐Golgi network (Figure S8C,D, Supporting Information). We postulated that the Sema3A protein might be secreted through encapsulation in microvesicles. To confirm this, we isolated vesicles from the supernatants of Ang II‐stimulated MiVECs and analyzed sEV‐associated protein markers in these vesicles. As revealed by iodixanol gradient density centrifugation, Sema3A containing vesicles floated in the middle of fractions 4 and 7, which contained the highest levels of classical sEV markers (Hsp70, TSG101, CD63) (Figure S9A–C, Supporting Information). Therefore, we selected these fractions for future experiments. To further confirm that Sema3A exists in sEVs, we isolated sEVs from the supernatants of MiVECs and performed immunoblotting, nanoparticle tracking analysis (NTA), and immunocolloidal gold staining. Immunoblotting of whole cell lysates (WCLs) and sEVs isolated from MiVECs demonstrated that sEVs revealed the presence of classical sEV markers, such as tetraspan CD63, TSG101, and Hsp70. In contrast, the sEVs presented an absence of expression of subcellular fractions, such as the endoplasmic reticulum marker GRP78 and Golgi‐matrix protein GM130 (Figure S9D, Supporting Information). The NTA yielded distributions that showed that the majority of particles ranged between 30 and 400 nm, with an average diameter of 120 nm (Figure S9E, Supporting Information). Immunogold electron microscopy analyses indicated gold‐labeled Sema3A accumulation at the membranes of disc‐shaped vesicles with a diameter of ≈100 nm (**Figure** **7**A). Next, we co‐stained Sema3A with CD63 and TSG101. Immunofluorescence co‐localization of Sema3A and CD63 suggested that Sema3A was encapsulated in sEVs (Figure S9F, Supporting Information). To further ascertain whether CD63 and Sema3A coexist on single sEVs, we stained sEVs with both CD63 and Sema3A antibodies, The results suggested the colocalization of CD63 with Sema3A in sEVs from both cell supernatants and human plasma (Figure 7B,C). Collectively, these data demonstrate that Sema3A is located in the membrane of isolated sEVs from MiVECs.

Upon confirming that Sema3A could be loaded into sEVs, we assessed the expression levels of Sema3A in sEVs isolated from Ang II‐treated or PBS MiVECs. As shown in Figure 7D, Sema3A was highly expressed in the cell lysates (WCLs) and sEVs from MiVECs following stimulation with Ang II, compared with the PBS control, while the levels of NRP1, CD63, and VEGFA showed no significant difference in sEVs (Figure 7D). To further validate the difference in Sema3A expression on the surface of sEVs, nano‐flow cytometry analysis was performed. The results showed that the expression of Sema3A expression on the surface of sEVs was higher in MiVECs treated with Ang II than in PBS MiVEC controls (Figure 7E).

It has previously been reported that the endosomal sorting complex required for transport (ESCRT) plays a critical role in sorting cargo proteins into sEVs. Next, we aimed to determine whether Sema3A directly interacts with the key protein component of TSG101 within the ESCRT. However, we did not observe a direct interaction between Sema3A and TSG101 (Figure S9G, Supporting Information). We observed that biotin‐labeled Sema3A was mostly engaged with late endosomes (Figure S9H, Supporting Information). This evidence also confirmed our assumption that Sema3A intended for secretion is transported through the sEV.

Next, we investigated whether Sema3A‐packaged sEVs caused capillary rarefaction. In brief, we examined the effect of blocking MiVECs‐sEV secretion on angiogenesis in vitro. As reported previously, Rab27a is necessary for the docking and fusion of multivesicular endosomes with the plasma membrane and is important for sEV secretion;^[^ ^19^, ^20^ ^]^ therefore, we knocked down Rab27a using shRNA in MiVECs to abolish sEV secretion. Next, we performed immunoblotting to analyze the expression of Sema3A and its related proteins in both MiVECs and culture media. The protein levels of Sema3A in whole‐cell lysates were dramatically upregulated after Rab27a knockdown (Figure 7F,G). *Sema3a* mRNA levels remained unchanged after Rab27a knockdown (Figure S9I, Supporting Information). However, it was significantly reduced in the culture medium (Figure 7H). Simultaneously, the expression of NRP1, SRF, and VEGFA was not affected by Rab27a knockdown (Figure 7F,G). More importantly, blocking the secretion of sEVs using Rab27a‐shRNA significantly increased the proliferation, migration, and tube formation of MiVECs (Figure S10A–C, Supporting Information). We also found that the blockade of sEV secretion by Rab27a knockdown reduced the interaction between NRP1 and Sema3A in MiVECs (Figure 7I). In contrast, the interaction between NRP1 and VEGFA was profoundly enhanced (Figure 7J). To further confirm whether the increase in Sema3A protein secretion from MiVECs was due to sEV trafficking, we knocked down Rab27a in MiVECs to abolish sEV secretion before overexpression of Sema3A; inhibition of sEV secretion significantly hindered Sema3A release into the extracellular space (Figure S11A,B, Supporting Information). Similarly, inhibition of sEV secretion attenuated the dysfunction of MiVECs induced by Sema3A overexpression upon Ang II stimulation (Figure S11C–E, Supporting Information). Overall, our findings confirmed that MiVECs predominantly secrete extracellular Sema3A in an sEV‐dependent manner and are involved in the dysfunction of angiogenesis.

Given that cardiac fibroblasts (CFs) are close neighbors of MiVECs in the perivascular region, we further investigated the impact of MiVEC‐derived sEVs on CFs (Figure S12A, Supporting Information). MiVEC‐derived sEVs were labelled with the lipophilic fluorescent dye PKH‐67 and incubated with CFs for 24 h. We observed that PKH‐67‐labeled sEVs were internalized and accumulated around the nucleus, suggesting uptake of sEVs by CFs (Figure S12B, Supporting Information). The CFs incubated with sEVs from MiVECs treated with Ang II showed significantly increased collagen‐related protein (Collagen I, Collagen III, and *α*‐SMA) expression compared to those treated with PBS control (Figure S12C,D, Supporting Information). Similarly, the secreted collagen I and collagen III in the CF conditional medium were upregulated after incubation with Ang II‐sEVs (Figure S12E, Supporting Information). Collectively, our results indicate that Sema3A‐packaged sEVs contribute to the switch from fibroblasts to myofibroblasts.

### 2.8 Semaphorin3A Deficiency Attenuated Overload‐Induced Cardiac Dysfunction and Capillary Rarefaction

**Figure 8:**
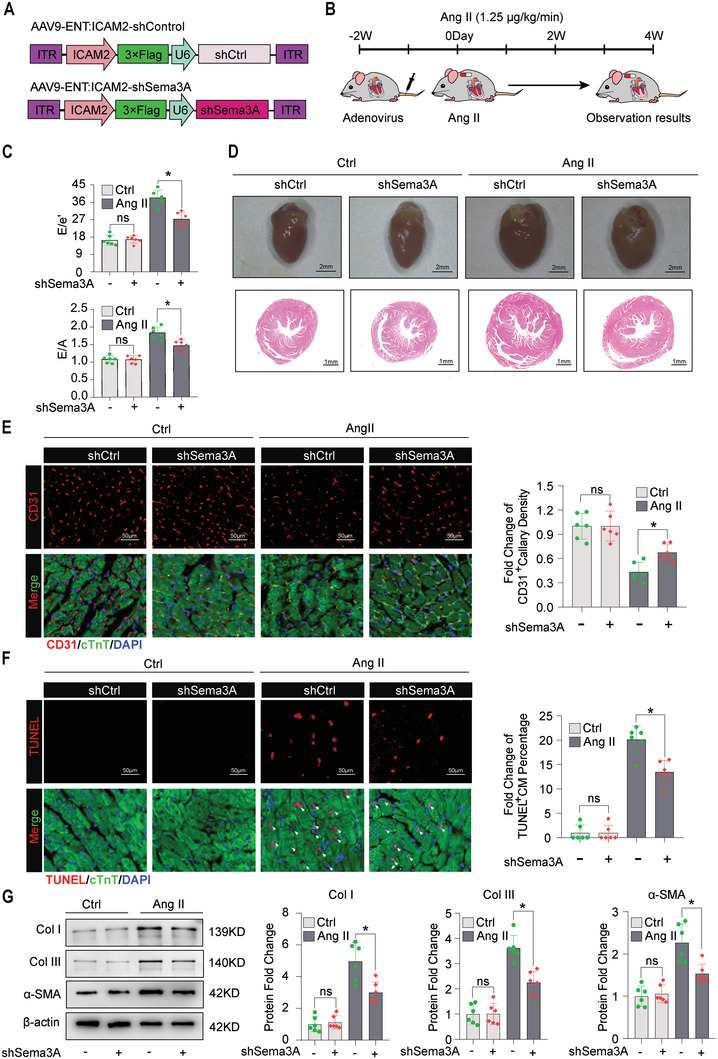
Sema3A deficiency attenuated pressure overload‐induced cardiac dysfunction and capillary rarefaction. A,B) Schematic illustration of the injection of AAV9‐ENT‐ICAM2 in vivo: Control (shCtrl) or AAV‐ENT‐ICAM2‐shSema3A (shSema3A) into mouse hearts. AAV‐ENT‐ICAM2: A serotype targeting vascular endothelial cells adeno‐associated virus with endothelial‐specific promoter ICAM2. C) Graphs showing the time course of the E/e' ratio (upper panel) and E/A ratio (lower panel). D) Gross morphology (upper panel, Scale bar = 2 mm) and histopathological sections stained with hematoxylin and eosin (H&E) (lower panel, Scale bar = 1 mm) of the shCtrl and shSema3A hearts after saline or Ang II infusion. E) Quantification of capillary density based on CD31 staining: red, CD31; green, cTnT; and blue, nuclei. Scale bar = 50 µm. *n* = 6 mice per group. F) Cardiomyocyte apoptosis assessed by TUNEL staining (red), cTnT marks cardiomyocytes (green), and DAPI marks nuclei (blue). Scale bar = 50 µm. *n* = 6 mice per group. G) Immunoblotting analysis of fibrosis‐related proteins (Collagen I, Collagen III, and *α*‐SMA) in AAV‐CON or AAV‐shSema3A hearts after infusion with saline control or Ang II (*n* = 6 mice per group). Data are presented as the mean ± SEM. *p < 0.05, by one‐way ANOVA. cTnT, cardiac troponin T; Col I, Collagen type1; Col III, Collagen type3.

We further explored the effects of Sema3A in MiVECs on capillary rarefaction. We constructed AAV9‐ENT vectors (based on adeno‐associated virus 9 (AAV9) serotype modification and enhanced the infection efficiency of vascular endothelial cells) carrying Sema3A‐shRNA under the ICAM2 promoter (**Figure** **8**A); we then, we delivered these AAV9‐ENT vectors through the tail veins of mice (Figure 8B). After 2 weeks, we performed fluorescence staining and immunoblotting to confirm the knockdown efficiency of Sema3A in MiVECs. Fluorescence staining of the flag indicated that most MiVECs were infected with AAV9‐ENT‐ICAM2 vectors (Figure S13A, Supporting Information); immunoblotting showed that Sema3A expression was profoundly reduced in MiVECs isolated from mice injected with AAV9‐ENT‐ICAM2‐shSema3A compared with those injected with AAV9‐ENT‐ICAM2‐control (Figure S13B, Supporting Information). While, Sema3A levels in isolated cardiomyocytes showed no significant difference (Figure S13B, Supporting Information).

We established an endothelial‐specific Sema3A knockdown mouse model using AAV vectors. We found that there was no difference between control and Sema3A knockdown mice in terms of cardiac function (Figure 8C) and capillary density (Figure 8E) before Ang II‐infusion, indicating that silencing of Sema3A does not affect basal cardiac function and capillary density in adult mice. Next, we investigated the potential role of Sema3A in pressure overload conditions, in which both control and Sema3A knockdown mice received continuous Ang II infusion for 4 weeks (Figure 8A). Despite the successful knockdown of Sema3A in MiVECs, we did not detect changes in the heart weight/body weight (HW/BW) (Figure S13C, Supporting Information), heart weight/tibia length (HW/TL) (Figure S13D, Supporting Information) ratios after cardiac pressure overload. However, wheat germ agglutinin staining showed that the cross‐sectional area of cardiomyocytes from Sema3A‐knockdown mice was smaller than that of control mice in response to pressure overload stimuli, implying that knockdown of Sema3A in MiVECs can prevent pressure overload‐induced hypertrophy (Figure S13E, Supporting Information). Furthermore, echocardiographic measurements showed improvement in left ventricular hypertrophy and diastolic function when Sema3A was knocked down in MiVECs upon pressure overload (Figure 8C).

Although Sema3A in MiVECs appears to play a limited role in hypertrophy in response to pressure overload, it may regulate the adaptive angiogenic response and interstitial fibrosis. Knockdown of Sema3A in MiVECs by injection of adenovirus carrying Sema3A shRNA and CD31 staining revealed greater capillary density in the hearts, when compared with mice that received adenovirus carrying control shRNA (Figure 8E). TUNEL staining results showed that the knockdown of Sema3A in MiVECs attenuated cardiomyocyte apoptosis induced by pressure overload (Figure 8F). Furthermore, immunoblotting analysis of fibrosis‐related proteins (collagen I, collagen III, and *α*‐SMA) showed that pressure overload‐mediated cardiac fibrosis was significantly reduced in endothelial‐specific Sema3A knockdown mice (Figure 8G). Overall, these data indicated that endothelial‐specific Sema3A knockdown attenuates pressure overload‐mediated microvascular rarefaction and cardiac fibrosis in vivo.

## 3 Discussion

Cardiac MiVECs serve as critical energy suppliers and effectors of angiogenesis.^[^ ^21^, ^22^ ^]^ Hence, oxygen and nutrients cannot be supplied abundantly for a compensatory increase in myocyte size (hypertrophy) during pressure overload when encountering insufficiencies in adaptive angiogenesis.^[^ ^23^, ^24^ ^]^ Although impaired angiogenic potential is known to be associated with microvascular rarefaction induced by pressure overload, the key mechanisms by which the maladaptive angiogenic response is induced are less understood. In the present study, we identified a pivotal role and novel platform for the increased Sema3A secreted from the sEVs of MiVECs to compete with VEGFA to binding to NRP1, and thus inhibit the adaptive angiogenic response during pressure overload.

In this study, the RNA sequencing of MiVECs isolated from Ang II‐infused and sham murine hearts was established. Sema3A was screened, and its expression was relativity upregulated, specifically in MiVECs, after heart pressure overload injury. Sema3A, a secreted protein, has previously been studied in the context of bone formation and neuronal development.^[^ ^18^, ^25^, ^26^ ^]^ However, the role of Sema3A in microvascular rarefaction remains unclear. Here, we identified Sema3A as a negative regulator of the angiogenic potential during Ang II‐induced pressure overload‐mediated microvascular rarefaction. Moreover, Sema3A was also found to be upregulated in TAC‐induced pressure overload, both in mouse heart tissue and serum, suggesting that the negative regulator of the angiogenic function of Sema3A may have broader implications for other pathological hypertrophic heart diseases.

The upregulation of Sema3A protein level was accompanied by an elevated *Sema3A* mRNA level, suggesting that regulation of Sema3A may occur at the transcriptional level. Bioinformatics‐based analysis predicted that SRF could bind to the *Sema3A* gene promoter, which was confirmed by luciferase reporter assay and ChIP analysis. Subsequently, we found that the knockdown of SRF decreased the expression of Sema3A, suggesting that SRF may act upstream of Sema3A, thereby regulating angiogenesis via transcriptional activation of *Sema3A* gene expression by directly binding to the promoter of Sema3A. After establishing a regulatory relationship between SRF and Sema3A, we investigated the role of SRF in angiogenic potential. In the current study, SRF was found to inhibit MiVEC proliferation, migration, and tube formation under Ang II conditions. Corroborating recent studies, SRF has been demonstrated to be a profibrotic molecule in hypertrophic remodeling and liver fibrosis.^[^ ^27^, ^28^, ^29^ ^]^ For instance, knockdown of SRF in hepatic stellate cells attenuated carbon tetrachloride‐induced hepatic fibrosis.^[^ ^30^, ^31^ ^]^ Other studies have shown that hypoxia‐induced upregulation of SRF impairs endothelial cell function.^[^ ^32^ ^]^ Collectively, these data support that SRF is a pathogenic mediator in fibrotic, remodeling, and hypertrophy‐associated processes in different types of cells.

NRP1 is a cell‐surface receptor^[^ ^33^, ^34^ ^]^ that is intimately involved in the development of the cardiovascular system,^[^ ^34^ ^]^ pathogenic angiogenesis,^[^ ^35^, ^36^ ^]^ and organogenesis outside the nervous system.^[^ ^33^, ^37^ ^]^ Previous studies have reported that NRP‐1 is predominantly expressed in the heart by endothelial cells, fibroblasts, and smooth muscle cells.^[^ ^38^ ^]^ NRP‐1 is typically manifested as a co‐receptor for Sema3A and VEGFA.^[^ ^39^ ^]^ Our data revealed that excessive Sema3A expression, induced by chronic pressure overload, leads to the interaction of Sema3A with NRP1 more closely and attenuates VEGFA‐mediated angiogenesis by competing with VEGFA for NRP1 binding, owing to which MiVECs lose their abilities of proliferation, migration, and tube formation, in the context of adaptive angiogenesis response. This evidence provides a novel and plausible mechanism, which indicates that microvascular rarefaction does not occur due to the lack of proangiogenic factors, and instead, NRP1 may be competitively blocked by the ligand whose expression is upregulated during pressure overload.

We explored the mechanisms underlying Sema3A secretion in MiVECs. Immunoelectron microscopy showed that Sema3A was localized in MVBs‐like vesicles. These data prompted us to speculate that Sema3A may be secreted through microvesicles. Immunoelectron microscopy and nano‐flow cytometry analyses indicated that Sema3A was located on the surface of sEVs, and interrupting sEV biogenesis profoundly reduced Sema3A release from the cytoplasm to the extracellular space. Collectively, our findings confirmed the hypothesis that sEVs act as vehicles of Sema3A secretion, and Sema3A was identified as a membrane protein in the sEVs of MiVECs. Consistent with our observations, findings from previous studies have reported VEGFA to be detected in sEVs.^[^ ^40^, ^41^ ^]^ Additionally, VEGFA was detected in the conditioned medium and in sEVs from MiVECs, which showed no significant difference compared to their levels in PBS control and Ang II‐stimulated groups. However, interruption of sEV biogenesis did not affect VEGFA protein secretion. One plausible explanation for this observation is that fundamental VEGFA trafficking does not occur through extracellular vesicles, and instead, it may occur via a direct gateway into the extracellular space.^[^ ^42^ ^]^

The roles of nucleic acids, including miRNAs, circRNAs, and DNA fragments, in sEVs are well characterized.^[^ ^15^, ^43^, ^44^ ^]^ For instance, in one of our previous studies, we showed that circular RNA Ube3a from M2 macrophage‐derived sEVs mediate myocardial fibrosis after acute myocardial infraction.^[^ ^43^ ^]^ However, it is unclear whether sEV proteins are involved in cardiac remodeling. In the present study, we identified Sema3A as a new member of sEV surface proteins and found that its expression was upregulated in MiVEC‐sEVs induced by pressure overload. Furthermore, our findings suggest that MiVEC‐derived Sema3A‐containing sEVs promote the expression of fibrosis‐related proteins, such as collagen I, collagen III, and *α*‐SMA, in cardiac fibroblasts and exacerbate fibrotic lesions. Thus, Sema3A‐positive sEVs may play a role in the crosstalk between injured MiVECs and cardiac fibroblasts during remodeling progression. However, a mechanistic explanation for the mediation of myocardial fibrosis by Sema3A‐positive sEVs in this study has not been established.

It should be noted that reduced vessel density leads to local hypoxia, oxidative stress, and inadequate perfusion, resulting in secondary cardiomyocyte hypertrophy and increased Sema3A secretion by MiVECs. This scenario creates a vicious cycle between hypertrophy and microvascular rarefaction in pressure overload‐induced cardiac remodeling induced by pressure overload. Here, the in vivo knockdown of Sema3A in MiVECs by shRNA‐mediated strategy confirmed the scientific significance and clinical relevance of Sema3A in the regulation of microvascular rarefaction. In a mouse model of pressure overload induced by Ang II infusion via osmotic minipumps, Sema3A knockdown in MiVECs showed a protective role in angiogenesis.

Overall, our study provides a conceptual advance in the understanding of the pathophysiology underlying microvascular rarefaction and provides a candidate therapeutic option for developing future strategies to mitigate capillary loss in pressure overload‐induced heart disorders.

## 4 Experimental Section

### Animals and Animal Care

C57BL/6J mice were obtained from the Shanghai Research Center of the Southern Model Organisms (Shanghai, China) and housed under specific pathogen‐free conditions in the animal center of Shanghai Medical College, Fudan University (Shanghai, China). All animals were fed a 12 h light/12 h dark cycle in a temperature‐controlled room with food and water ad libitum. All animal experiments were performed in accordance with the NIH guidelines (Guide for the Care and Use of Laboratory Animals) and were conducted in accordance with protocols approved by the Animal Care and Utilization Committee of Zhongshan Hospital affiliated with Fudan University (No. 2022–641).

### Protein Extraction and Immunoblotting

Snap‐frozen cardiac tissues and isolated cells were homogenized in RIPA solution (Beyotime, Shanghai, China) containing a protease inhibitor cocktail. Protein concentrations were determined using the BCA Protein Assay kit (Thermo Fisher Scientific, MA, USA). Equal amounts of protein extracts were resolved through SDS‐PAGE and then transferred onto nitrocellulose (NC) membranes. The membranes were then blocked using western blocking solution (Beyotime, #P0023B, Shanghai, China) and incubated with the desired primary antibodies at 4 °C overnight. The membrane was washed and incubated with the appropriate secondary antibodies at room temperature for 1 h. Immunoreactivity was visualized using the ChemiDoc Imaging System (Bio‐Rad, CA, USA). The dilution and detection of the primary antibodies are summarized in Table S1, Supporting Information.

### Collection of Myocardial Hypertrophy Samples

Myocardial hypertrophy samples were collected during valve replacement (AVR) and control heart samples were collected from unmatched or rejected healthy donor hearts. Written informed consent was obtained from the families of prospective heart donors before sample collection. All experiments were collected and approved by the Ethics Committee of Zhongshan Hospital affiliated with Fudan University (Approval No.2022‐267R) and conformed to the principles outlined in the Declaration of Helsinki.

### Quantitative Reverse Transcription Polymerase Chain Reaction

Total RNA was extracted using a TRIzol kit (Invitrogen, USA) according to the manufacturer's instructions. Total RNA (1 µg) was reverse‐transcribed into cDNA using the PrimeScript RT kit (TaKaRa, #RR036A). Real‐time PCR (qPCR) analysis was performed using CFX96 real‐time system (Bio‐Rad Laboratories, Inc., Hercules, CA, USA) using a SYBR Premix Ex TaqTM kit (TaKaRa, #RR420A). *β*‐actin was used for internal normalization, and relative gene expression was calculated using the 2−ΔΔCt method. The primers used are listed in Table S2, Supporting Information.

### Enzyme‐Linked Immunosorbent Assay

Collagen I and Collagen III concentrations in the medium were measured with ELISA kits (R&D, USA), while Sema3A contents in sera and medium were measured with ELISA kits (Cusabio, Nanterre, France), following the manufacturer's instructions.

### Immunofluorescence Staining

Immunofluorescence staining was performed on tissue sections and cells as described previously.^[^ ^45^ ^]^ After deparaffinization, rehydration, and heat‐mediated antigen retrieval with citrate buffer (10 mm) at pH 6.0, the tissue sections were blocked with goat serum (Beyotime, Shanghai, China). The sections were then incubated with primary antibodies at 4 °C overnight followed by washing and incubation with the secondary antibodies for 1 h at room temperature. Nuclei were counterstained with 4',6‐diamidino‐2‐phenylindole (DAPI; Beyotime, Shanghai, China). Confocal images were obtained using the Olympus FV‐1000 confocal microscope, and the acquired images were processed using the Olympus Fluoview Software (Olympus, Tokyo, Japan). The primary and secondary antibodies used in this study with the dilution factors are listed in Table S1, Supporting Information.

Cells were washed with pre‐cooled PBS, fixed with 4% paraformaldehyde, permeabilized with 0.1% Triton X‐100, and blocked with goat serum. The subsequent procedure was the same as that used for immunostaining of paraffin sections.

### EdU‐Based Cell Proliferation Assay

EdU assay (RiboBio, Guangzhou, China) was performed according to the manufacturer's instructions. Briefly, cells were treated under predetermined conditions, seeded on confocal dishes at 1 × 10^6^ per well and incubated with EdU solution (50 µM) for 2 h, fixed with 4% paraformaldehyde for 30 min, decolorized with glycine (2 mg mL^−1^), permeabilized with 0.5% Triton X‐100, and washed three times with pre‐cooled PBS. The cells were then treated with 1× Apollo staining reaction solution (100 µL) for 30 min in the dark. The DNA was incubated with Hoechst (1:100 dilution) for 30 min. The images were visualized using a fluorescence microscope (Leica DMI8, Wetzlar, Germany), and the results were analyzed using ImageJ software^[^ ^46^ ^]^ (ImageJ, National Institutes of Health, USA).

### Transwell Migration Experiment

Cell migration experiments were performed in a 24‐well transwell chamber (3422, 8‐µm pore size; Costar, USA). Briefly, cells were subjected to different experimental conditions, prepared into cell suspensions containing 0.1% FBS (1 × 10^4^ per well) and inoculated in the upper chamber. Then, medium (500 µL) containing 10% FBS was added to the lower chamber of the transwell, and the plate was placed in an incubator at 37 °C with 5% CO2. The chambers were removed, and the unmigrated cells in the chambers were gently wiped off with cotton swabs. Cells were fixed with 4% paraformaldehyde for 30 min, stained with 0.2% crystalline violet for 15 min, counted in five random fields under a microscope, and photographed.

### Tube Formation Assay

Pre‐melted matrigel (BD Biosciences, #356231) was inoculated into a 24‐well plate at 150 µL per well, and incubated at 37 °C in a 5% CO2 incubator for 1 h until the Matrigel solidified. Then, cell suspension (200 µL) (≈1 × 10^5^ cells) was added to each well. After incubation at 37 °C for 6 h, the images were obtained using a fluorescence microscope.

### Statistical Analysis

All statistical analyses were performed using GraphPad Prism 8.0 (San Diego, CA, USA) and SPSS 21.0 statistical software packages (IBM, Armonk, NY, USA). All data are presented as mean ± SEM. Normal distribution of data was assessed using the Shapiro‐Wilk normality test. Statistical comparisons between two groups were determined through the unpaired, 2‐tailed Student *t*‐test, and one‐way analysis of variance was used to compare multiple groups. Values of *p* < 0.05 were considered to be statistically significant. All experimental *n* numbers are provided in the figure legends.

Additional Section 4 is available in online Supporting Information.

## Conflict of Interest

The authors declare no conflict of interest.

## Supporting information

Supporting Information

## Data Availability

The data that support the findings of this study are available from the corresponding author upon reasonable request.
