# Supplementary material for: Semaphorin3A Exacerbates Cardiac Microvascular Rarefaction in Pressure Overload‐Induced Heart Disease
Source: Adv Sci (Weinh). 2023 Jun 13;10(21):2206801. doi: 10.1002/advs.202206801 (PMC10375119; doi:10.1002/advs.202206801)
Supplement: Supplementary file 1 — Supporting Information [file ADVS-10-2206801-s001.pdf]

ADVANCED SCIENCE
Open Access

Supporting Information

for *Adv. Sci.*, DOI 10.1002/advs.202206801

Semaphorin3A Exacerbates Cardiac Microvascular Rarefaction in Pressure Overload-Induced Heart Disease

Chaofu Li, Yongchao Zhao, Fuhai Li, Zimu Wang, Zhimei Qiu, Yukun Yang, Weidong Xiong, Rui Wang, Han Chen, Fei Xu, Tongtong Zang, Zhiqiang Pei, Yan Wang, Bei Shi, Li Shen* and Junbo Ge**

Supporting Information

Semaphorin3A Exacerbates Cardiac Microvascular Rarefaction in Pressure Overload–Induced Heart Disease

Chaofu Li, Yongchao Zhao, Fuhai Li, Zimu Wang, Zhimei Qiu, Yukun Yang, Weidong Xiong, Rui Wang, Han Chen, Fei Xu, Tongtong Zang, Zhiqiang Pei, Yan Wang, Bei Shi, Li Shen*, and Junbo Ge**

Dr. C. Li, Dr. Y. Zhao, Dr. Z. Wang, Dr. R. Wang, Dr. H. Chen, Dr. F. Xu, Dr. T. Zang, Dr. Z. Pei, Prof. L. Shen, Prof. J. Ge
Department of Cardiology
Zhongshan Hospital
Fudan University
Shanghai Institute of Cardiovascular Diseases
180 Fenglin Road, Xuhui District, Shanghai 20032, P. R. China
E-mail: shen.li1@zs-hospital.sh.cn; jbge@zs-hospital.sh.cn

Dr. F. Li
Department of Cardiology
Affiliated Hospital of Qingdao University,
Qingdao, 266000, China

Dr. Z. Qiu, Dr. W. Xiong, Dr. Y. Wang, Prof. B. Shi,
Department of Cardiology
Affiliated Hospital of Zunyi Medical University
Zunyi, 563000, China;
E-mail: shib@zmu.edu.cn

Dr. Y. Yang
The neuroscience lab,
University Hospital Essen,
University of Duisburg-Essen,
Essen D-45122, Germany

Supplemental Table 1. Antibodies used in this study

Antibody specificity	Manufacture	Cat. No.	Species	Dilution
Primary antibodies for western blot				
Anti-Semaphorin 3A	Abcam	ab199475	Rabit	1:1000
HRP-conjugated β-Actin	Proteintech	HRP66009	Mouse	1:5000

Anti-Serum Response Factor	Proteintech	66742-1-Ig	Mouse	1:4000
Anti-Histone-H3	Proteintech	17168-1	Rabit	1:5000
Anti-Neuropilin 1	Abcam	ab81321	Rabbit	1:1500
Anti-VEGFA	Abcam	ab214424	Rabbit	1:1000
Anti-GM130	Abcam	ab52649	Rabbit	1:1000
Anti-GRP78	Abcam	ab21685	Rabbit	1:1000
Anti-HSP70	CST	4872T	Rabbit	1:2000
Anti-TSG101	Proteintech	28283-1	Rabbit	1:5000
Anti-CD63	Abcam	ab134045	Rabbit	1:1000
Anti-Rab27a	CST	69295S	Rabbit	1:1000
Anti-FLAG-tag	Sigma	F1804	Mouse	1:2000
Anti-Myc-tag	Proteintech	60003-2-Ig	Mouse	1:10000
Anti-Collagen I	Abcam	ab138492	Rabbit	1:2000
Anti-Collagen III	Proteintech	22734-1	Rabbit	1:500
Anti-α-SMA	Proteintech	14395-1	Rabbit	1:4000
Secondary antibodies for western blot				
HRP-conjugated goat anti-mouse IgG	Proteintech	SA00001-1	Goat	1:5000
HRP-conjugated goat anti-rabbit IgG	Proteintech	SA00001-2	Goat	1:5000
Antibody for ChIP, Co-IP and PLA				
Anti-Serum Response Factor	Abcam	ab252868	Rabbit	1:100
Anti-Semaphorin 3A	Abcam	ab199475	Rabbit	1:50
Anti-TSG101	Proteintech	28283-1	Rabbit	1:100
Anti-VEGFA	Abcam	ab1316	Mouse	1:200
Anti-Neuropilin 1	Abcam	ab81321	Rabbit	1:30
Primary Antibodies for Immunofluorescence				
Anti-Cardiac Troponin T	Abcam	ab8295	Mouse	1:500
Anti-ACTN2	Sigma	A7811	Mouse	1:1000
Anti-CD31	Abcam	ab222783	Rabbit	1:150
Anti-Vimentin	Proteintech	10366-1	Rabbit	1:300
Anti-CD45	Proteintech	60287-1-Ig	Mouse	1:300
Anti-Semaphorin 3A	Santa-Cruz	sc-74555	Mouse	1:300
Anti-CD63	Abcam	ab217345	Rabbit	1:200
Anti-TSG101	Proteintech	28283-1	Rabbit	1:300
FITC-labeled WGA	Sigma	L4895	NA	1:200
Secondary antibodies for Immunofluorescence				
Anti-mouse IgG H&L (Alexa Fluor® 488)	Abcam	ab150113	Goat	1:500
Anti-rabbit IgG H&L (Alexa Fluor® 594)	Abcam	ab150080	Goat	1:600
Rhodamine-conjugated Anti-rabbit IgG	Proteintech	SA00007-2	Goat	1:100

Antibodies for flow cytometry			
Anti- mouse -vWF-FITC	BIOSS	bs-0586R	1:200
Anti- mouse -CD31- FITC	BioLegend	W18222B	1:150
Anti - Sema3A -FITC	BIOSS	bs-1121R	1:200
FITC-Isotype Ctrl Antibody	BioLegend	400633	1:150

Supplemental Table 2. Primers for RT-qPCR used in this study

Gene	Species	Sequence 5'-3'	
Sema3A	Mouse	F	GAAGAGCCCTTATGATCCCAAAC
		R	AGATAGCGCAAGTCCCGTCCC
SRF	Mouse	F	GTGCCACAGTATGGTCGTTG
		R	ACCCCCATTCCTGTATCTCC
Meox2	Mouse	F	AATCTAGACCTCACTGAAAGACAGG
		R	CTTGCTGTCCCCCTTTGA
HSF2	Mouse	F	ATCCCTTTGGAAGGAGGTGT
		R	TCACAAGTTGATTATTCTGAACCAA
TEAD4	Mouse	F	ATCCTGACGGAGGAAGGCA
		R	GCTTGATATGGCGTGCGAT

Supplemental Table 3. List of the sequences of shRNAs

Gene Symbol	NO.	Species	Sequence
shSema3A	#1	Mouse	CGAGACTTCATGCAGCTCATT
	#2	Mouse	CCCAGTGTTTCCTATAAATAA
	#3	Mouse	GCCTTGGTATATTGGCAATTT
shSRF	#1	Mouse	GATGGAGTTCATCGACAACAA
	#2	Mouse	GCCATGTGTATACCTTTGCCA
	#3	Mouse	AGCAGCAACCTCACCGAGCTA
shNRP1	NA	Mouse	CCTGCTTTCTTCTCTTGGTTT
shRab27	NA	Mouse	GCTTCTGTTCGACCTGACAAA

SUPPLEMENTARY MATERIALS AND METHODS

Application of angiotensin II via osmotic minipump implantation

C57BL/6N mice (14-16 weeks, average body weight of 25–30 g) were used for angiotensin II (Ang II) experiments. Briefly, mice were anesthetized with 2% isoflurane and implanted with a subcutaneous osmotic micropump (AZLET; Cupertino, CA, USA) containing sterile saline NaCl (0.9%) or Ang II (1.25 μg/kg/min) and dissolved in sterile saline, as described previously [1].

Isolation and culture of cells from adult mouse hearts

Heart cells were isolated through an enzymatic and mechanical dissociation procedure, as described previously [2, 3], with a modification of the previous protocol. C57BL/6J mice were anesthetized with an intraperitoneal injection of pentobarbital (50 mg/kg), and the chest was opened to expose the heart and cut off the descending aorta and inferior vena cava. Subsequently, EDTA buffer (7 mL) of was rapidly injected into the base of the right ventricle to flush the heart. The hearts were then transferred to a fresh EDTA buffer. Digestion was achieved by successively injecting EDTA buffer (10 mL), perfusion buffer (3 mL) and collagenase buffer (30-50 mL) into the left ventricle (LV). The centrifugal chamber and ventricle were separated, and forceps were used to pull the ventricle into 1 mm pieces. The cells were dissociated by gently grinding the cell debris, and then, stop buffer (5mL) was added to terminate the digestion process. The cells were filtered through a 100 μm filter and then collected. CMs were purified using two consecutive rounds of gravity sedimentation. Cells were seeded in a petri dish, which was pre-plated with lamin. In the supernatant containing non-myocyte cells, magnetic separation of $CD45^+$ white blood cells, MiVECs were gathered using CD31-coupled microbeads (Thermo Fisher, USA) and further cultured in endothelial culture medium (ECM, ScienCell, USA). Cardiac fibroblasts were collected using MEFSK4-coupled microbeads and further cultured in DMEM complete medium.

Clinical Evaluation of Subjects

General data, including sex, age, and duration of chronic heart failure, were collated for all subjects. Body mass index (BMI) was calculated from the measured weight and height according to the formula, BMI = weight/height2 (kg/m^2). After the participants rested for 10 min, two sitting blood pressure measurements were taken using an automated sphygmomanometer with an appropriate cuff size, and the mean value was recorded as the final blood pressure value. Fasting blood samples were collected to determine the plasma Sema3A levels. Plasma Sema3A protein levels were quantified using ELISA according to the manufacturer's instructions (Jianglai Biotechnology,

Shanghai China, #JL19924-96T).

Nuclear-cytoplasmic fractionation

For nucleocytoplasmic separation, samples were prepared using the NE-PER Nuclear and Cytoplasmic Extraction Kit (Thermo Fisher Scientific, MA, USA) according to the manufacturer's instructions. Briefly, the cells were pelleted and resuspended in lysis buffer (0.5% NP40, 150 mM NaCl, 50 mM Tris-HCl pH 7.4) and incubated on ice for 5 min. The lysate was then centrifuged (16,000 × *g* at 4 °C for 10 min), resulting in separation of the nucleoplasm and cytoplasmic fractions. The nuclear pellets were washed twice with wash buffer, resuspended in fractionation buffer, and incubated on ice for 15 min. Next, the nucleoplasmic fraction was separated by centrifugation at 16,000 × *g* at 4 °C for 10 min and analyzed through western blotting.

Cell viability assay

Cell viability was determined using the Cell Counting Kit 8 (Dojindo Laboratories, Kumamoto, Japan), according to the manufacturer's instructions, as described previously. The OD values were measured at 450 nm using a microplate reader (Thermo Fisher Scientific, Carlsbad, CA, USA).

Lentivirus-mediated gene knockdown

SRF, *Sema3A*, *NRP1*, and *Rab27a* knockdowns were generated using a lentivirus-mediated delivery system. Plasmids carrying the target gene shRNA and a negative control (shRNA-Ctrl) were designed and synthesized by Genechem Company (Shanghai, China). All viruses were packaged in 293 T cells, according to the manufacturer's instructions. Cells were treated as indicated and infected with lentivirus according to the manufacturer's instructions. At the end of the transfection experiment, the transfection medium was replaced with fresh medium for the subsequent experiments.

Plasmid transfection

Plasmid transfection was performed using the Lipofectamine 3000 transfection

reagent (Invitrogen). Briefly, plasmid DNA (4 μg per well) in six-well dishes and Lipofectamine 3000 were mixed with Opti-MEM at a total volume of 500 μL (Life Technologies) and incubated for 20 min at room temperature. Next, the DNA–Lipofectamine complex was added to the cells in transfection media. After 3 h of incubation, the medium was refreshed for an additional 48 h and the cells were collected for analysis.

Chromatin immunoprecipitation (ChIP)

Chromatin-immunoprecipitation (ChIP) was performed using an assay kit following the manufacturer's instructions (Millipore, #17-295). Briefly, the samples were crosslinked with 1% formaldehyde at room temperature for 10 min, and crosslinking was terminated with glycine (125 mM). The samples were then sedimented, washed, and placed on ice for 30 min with ChIP lysis buffer. The lysates were sonicated to reduce the DNA length from 200 to 1,000 bp. Chromatin samples were incubated with an antibody against SRF and normal rabbit IgG overnight at 4 °C, followed by incubation with Protein A Dynabeads at 4 °C for 4 min. The precipitated DNA was purified using a spin column. qRT-PCR was performed using primers specific for the Sema3A promoter (listed in Appendix Table S3).

Luciferase reporter assay

The luciferase assay was performed using a dual luciferase assay system kit (Promega, USA), according to the manufacturer's protocol. The full-length, truncated segments and mutant promoter of the Sema3A gene were cloned into the pGL3 basic vector (Genecreate, Wuhan, China) and co-transfected with the SRF empty vector or overexpression vector. After 48 h of incubation, luciferase activity was measured using a dual-luciferase reporter assay system (Promega, USA).

Co-immunoprecipitation (Co-IP)

Co-immunoprecipitation (Co-IP) was performed using a magnetic IP kit (Thermo Fisher Scientific, Carlsbad, CA). Briefly, after the samples were harvested, they were

lysed at 4 °C in ice-cold IP lysis buffer for 30 minutes and then centrifuged (12,000 × *g*, 10 minutes), following which equal volumes of lysates were incubated with various irrelevant IgG or specific antibodies and crosslinked onto the magnetic beads with rotation for 1 hour at room temperature. After removing the unbound sample, protein A/G magnetic beads were washed extensively with IP wash buffer, and the proteins were eluted by boiling in 1× SDS sample buffer before SDS-PAGE.

In situ proximity ligation assay (PLA) assay

Proximity ligation assays (PLA) were used to evaluate the protein interactions of NRP1-Sema3A (Red Starter kit) and NRP1-VEGFA (Green Starter kit). The cells were washed, fixed, permeabilized, and blocked as described for the previous immunofluorescence staining process. The two primary antibodies raised in different species were then incubated with the samples overnight. The proximity ligation reaction and signal visualization were performed following the manufacturer's instructions (Duolink in situ PLA Detection Kit, Sigma-Aldrich). PLA signals were visualized and images were taken using an Olympus FV-1000 confocal microscope (Olympus, Tokyo, Japan). The number of PLA signals per cell was quantified using the Duolink Image Tool software (Sigma Aldrich).

sEVs purification and characterization

sEVs were isolated from the conditioned medium through differential ultracentrifugation (Supplementary Figure 9A), as described previously [4, 5]. Conditioned media were subjected to differential centrifugation (300 × *g* for 10 minutes, 2,000 × *g* for 10 min) to remove cell debris and dead cells, and the resulting supernatant was filtered through a 0.22-μm filter. The filtered supernatant was ultracentrifuged at 100,000 × *g* (SW 40Ti rotor; Beckman Coulter Optima XPN 90) for 4 h at 4 °C. In addition, we examined the characteristics of sEVs using iodixanol gradient centrifugation, as previously reported [4]. Briefly, isolated sEVs were layered on top of a discontinuous iodixanol gradient and centrifuged at 100,000 × *g* for 18 h. The resulting fractions and their densities were measured at 340 nm based on the

absorbance values from the standard curve, to estimate the density of each fraction collected from the samples.

Nanoparticle tracking analysis

Small extracellular vesicle concentrations and particle sizes were determined by nanoparticle tracking analysis (NTA; particle Metrix, Meerbusch, Germany) and its corresponding software (ZetaView 8.02.31).

Nano flow cytometry

Nano flow cytometry (nFCM) uses specialized equipment to apply the fundamentals of standard flow cytometry to submicron particles [6]. Nanoflow cytometry was performed as described previously [7]. Briefly, before EV measurements, optical alignment of the nFCM system was performed using monodisperse silica nanoparticles, and the samples were diluted to a concentration of approximately 10^9 particles/mL to avoid swarm detection. The other steps for nFCM followed routine flow cytometry procedures.

Electron microscopy and immunogold labeling

Samples were fixed using 4% paraformaldehyde, 0.2% glutaraldehyde in sodium phosphate buffer (200 mM) for 6 h at 4 °C on a formvar-carbon-coated electron microscope grid. These samples were then incubated with 0.1% bovine serum albumin and 10% normal donkey serum (20 min), followed by incubation with anti-Sema3A antibodies. Normal IgG was used as a negative control. After extensive washes, the cells were incubated with secondary antibodies conjugated with 10 nm gold particles. The grid was washed with PBS, fixed in 2% glutaraldehyde for 5 min, rinsed in distilled water, stained with 2% aqueous uranyl acetate[8], and examined using a Hitachi H600 TEM (H600, Hitachi, Japan) at 75 kV.

Subcellular fractionation

Subcellular fractionation was performed, as described previously [9]. Briefly, cells were pelleted and suspended in cold cytoplasmic lysis buffer (10 mM HEPES, 10 mM

NaCl, 1 mM KH2PO4, 5 mM NaHCO3, 5 mM EDTA, 1 mM CaCl2, 0.5 mM, MgCl2). After incubation on ice for 5 min, the cells were layered onto cold sucrose buffer and centrifuge at 6,300 × *g* for 10 min at 4 °C. The resulting pellet was resuspended in ice-cold TSE buffer (10 mM Tris, 300 mM sucrose, 1 mM EDTA, 0.1% IGEPAL-CA 630 (v/v), pH 7.5) and further lysed using a homogenizer (4,000 × *g* for 5 min). The resulting pellet was washed twice with TSE buffer. The final pellet (nucleus) was resuspended in TSE buffer.

Sucrose density gradient centrifugation

Sucrose gradient centrifugation was performed, as described previously [10]. Briefly, the cells were pelleted and homogenized in lysis buffer (10 mM Tris–HCl, 1 mM EDTA, pH 7.4). Subsequently, the homogenate was loaded onto the step gradient sucrose (layers of 2, 1.3, 1.16, 0.8, 0.5, and 0.25 M sucrose) and centrifuged at 100,000 × *g* for 2.5 hours. Fractions were collected from the top of the gradient and analyzed by western blotting.

Transthoracic echocardiography

Mouse transthoracic echocardiography was performed using a high-frequency ultrasound system (Visualsonics Vevo 2100, Toronto, Canada). The mice were initially anesthetized with 3% isoflurane and maintained under anesthesia with 1.5% isoflurane during ultrasound image acquisition. The heart rate during image acquisition was maintained in the range of 450–550 bpm. Systolic and diastolic function were assessed, as described previously [11].

Histological analysis

Heart samples were harvested and washed in pre-cooled physiological saline, immediately fixed in 4% paraformaldehyde at 4 °C for at least 48 h, and embedded in paraffin. 4-μm-thick sections were cut. Sections were stained with hematoxylin and eosin (Leica Microsystems, Germany) or immunohistochemically. Cardiomyocyte cross-sectional area was determined by staining with fluorescein isothiocyanate-

conjugated WGA (Sigma-Aldrich, # L4895) and quantified using Image-Pro Plus software (version 6.0); WGA wheat germ agglutinin.

Terminal dUTP nick end-labeling (TUNEL) staining

To detect myocardial apoptosis, TUNEL staining of heart sections was performed using an In Situ Cell Death Detection Kit (TMR red, Roche). Myocyte cytoplasm was marked with cTnT and nuclei were stained with DAPI. TUNEL staining was performed according to the manufacturer's instructions.

Injection of adeno-associated virus vectors

Adeno-associated virus 9 (AAV9) particles were generated as described previously, with some modifications. In other words, a heptapeptide SLRSPPS was inserted through the AAV9 capsid A589 site, which can significantly increase the infection efficiency of endothelial cells[12]. AAV viral vectors were delivered by tail vein injection. Mice were placed in a fixator to fix the head and limbs. Before injection, the mice were exposed to a heat lamp to sufficiently dilate the tail vein, and viral injections were performed using an insulin syringe (Microfine, 29 G, U-50, BD Biosciences).

SUPPLEMENTARY FIGURES AND FIGURE LEGENDS

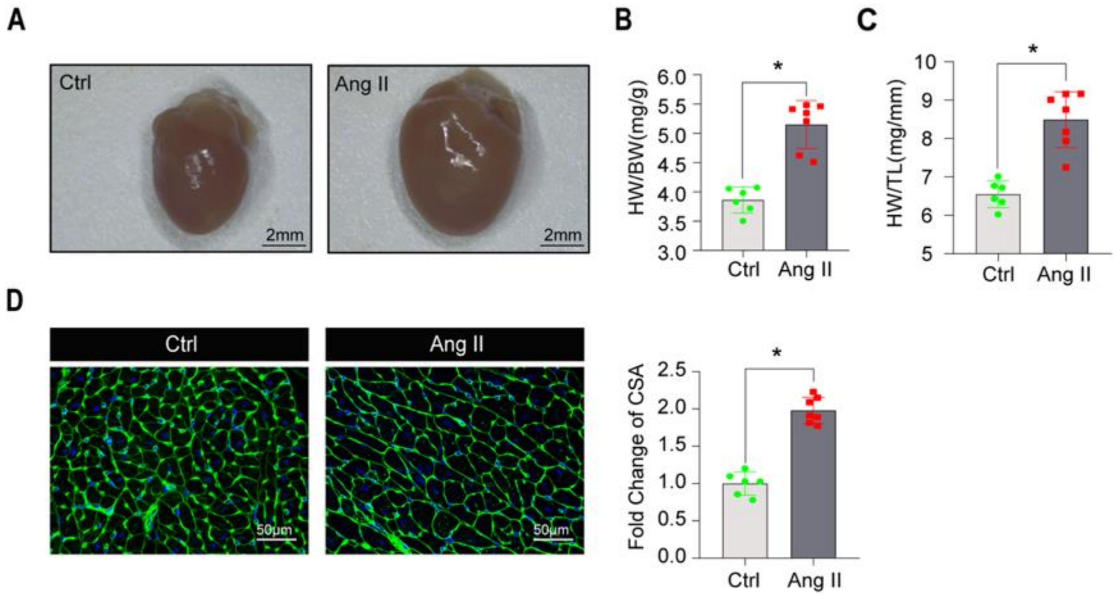

Supplemental Figure S1. Stablished Ang II-induced mouse model of cardiac remodeling.

A, Representative morphologies of the hearts from the saline control and Ang II-treated groups. Scale bar = 2 mm. **B-C**, Heart weight/body weight (HW/BW) and heart weight/tibia length (HW/TL) ratios of saline control and Ang II-treated mice. n = 6 mice per group. **D**, Heart sections were stained with WGA-FITC (green) to demarcate cell boundaries. Scale bar=50 μm. n = 6 mice per group. For all statistical plots, the data are presented as the mean ± SEM. *$P < 0.05$ between the two indicated groups by 2-tailed Student's *t*-test. Ang II, angiotensin II; WGA, wheat germ agglutinin; FITC, fluorescein isothiocyanate; CSA, mean cross-sectional area.

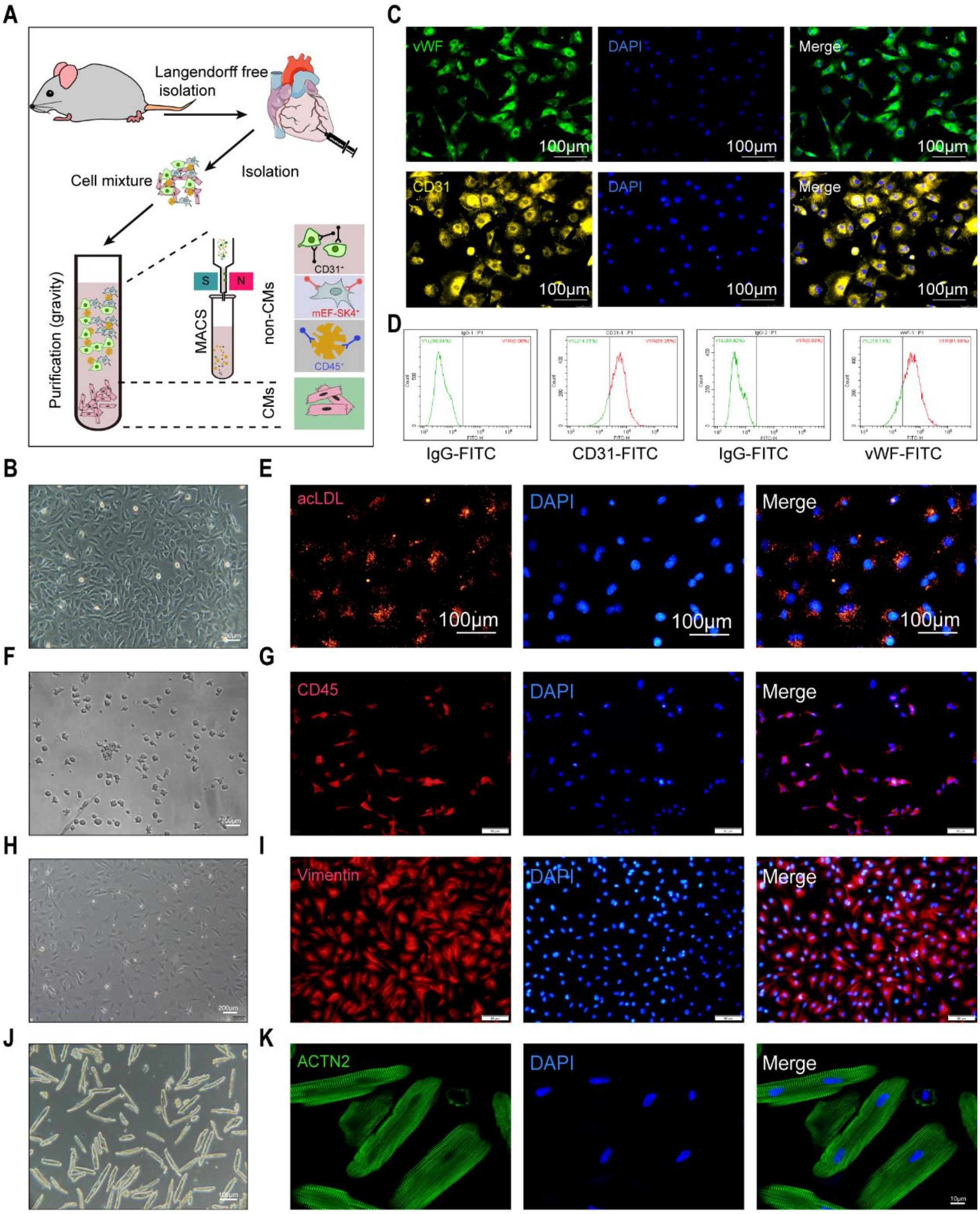

Supplemental Figure S2. Isolation of myocardial microvascular endothelial cells.

A, Schematic outline of myocardial microvascular endothelial cell (MiVECs) purification. **B**, Cobblestone morphology of isolated MiVECs, scale bar = 200 µm. **C**, Representative immunostaining for endothelial cell surface markers vWF (green) and CD31 (yellow) in MiVECs. The nuclei were stained blue with DAPI. Scale bar = 100 µm. **D,** Representative images of flow cytometry histograms showing endothelial cell surface marker expression in isolated MiVECs. **E**, Endothelial functional assays: Uptake of Dil-Ac-LDL by immunofluorescence (red, Dil-Ac-LDL; blue, DAPI);

Scale bar = 100 μm. **F-G**, Representative images of light microscopy (**F**) (scale bar = 200 μm) and immunostaining (**G**) (scale bar = 50 μm) for $CD45^+$ cells. **H-I**, Phase-contrast image of isolated cardiac fibroblasts (**H**) (scale bar = 200 μm) and immunostaining for vimentin (**I**) (scale bar = 50 μm). **J-K**, Phase-contrast image of isolated cardiac myocytes (**J**) (scale bar = 100 μm) and immunologic staining with ACTN2 antibody (**K**) (scale bar = 10 μm). vWF indicates von willebrand factor; DAPI, 4′,6-diamidino-2-phenylindole; Dil-Ac-LDL, dil-acetylated low-density lipoprotein; ACTN2, sarcomeric-α-actinin.

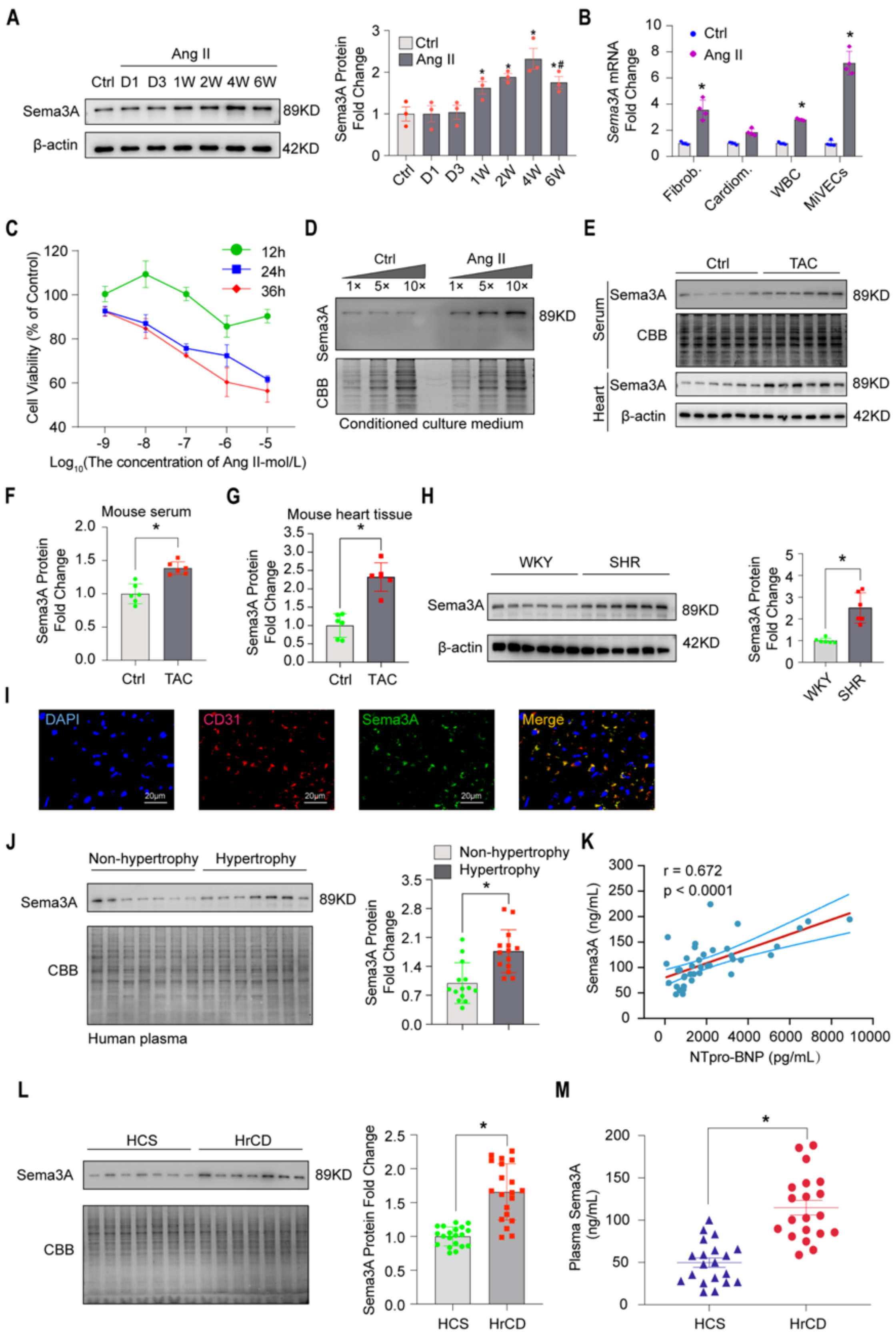

Supplemental Figure S3. Expression of Sema3A.

A, Representative immunoblotting analysis of Sema3A protein expression at set time points (1 d, 3 d, 1 w, 2 w, 4 w, and 6 w) in Ang II–infused mouse hearts (peak at 4

weeks). The expression levels were normalized to that of β-actin. Data were obtained from three independent experiments. *P < 0.05, versus saline control group (Ctrl), $^{\#}P$ < 0.05, versus 4 weeks group (4 W); n = 3 mice per group. **B** RT-qPCR analysis of the expression level of Sema3A in cardiac fibroblasts (Fibrob.), cardiomyocytes (Cardiom.), white blood cells (WBC), and MiVECs were isolated directly from the heart. *P < 0.05, versus saline control group (Ctrl); n = 4 mice per group. **C**, Concentration-time curve of MiVEC viability induced by Ang II; Ordinate, relative cell viability (% of control); abscissa, log10(Ang II concentration, mol/L); MiVECs were treated with Ang II (0.001, 0.01, 0.1, 1, and 10 μM) for 12, 24, and 36 h. The CCK-8 assay showed that Ang II caused a time- and concentration-dependent decrease in the viability of MiVECs. **D**, Immunoblotting analysis of Sema3A from MiVECs with or without Ang II treatment (concentrated 1×, 5×, or 10×). Coomassie blue staining shows total proteins on the gel. **E-G**, Sema3A protein levels in mouse serum (**F**) and heart (**G**) were detected by immunoblotting 4 weeks after TAC or sham surgery, respectively; n = 6 mice per group. **H**, Sema3A expression in myocardial tissues of Wistar Kyoto (WKY) and spontaneously hypertensive rats (SHRs). Protein expression was quantified and normalized to that of β-actin (n = 6 mice per group). **I**, Double immunofluorescence staining of Sema3A (green) and CD31 (red) in the hearts of hypertrophic patients; nuclei stained with DAPI (blue), scale bar = 20 μm. **J**, Representative Immunoblotting and quantification showing Sema3A expression levels in heart samples from non-hypertrophic (n = 14) and hypertrophic patients (n = 14). **K**, Correlation between circulating Sema3A and NT-proBNP levels (Spearman correlation, r = 0.672). **L**, Representative Immunoblotting and quantification showing Sema3A expression levels in serum from patients with hypertension-related cardiac dysfunction (n = 20) and healthy control subjects (n = 20). Blots stained with Coomassie brilliant blue served as a loading control. **M**, Sema3A serum concentration (pg/mL) measured by ELISA， n = 20. All data are presented as mean ± SEM; *P < 0.05; one-way ANOVA was performed in **A**, Two-way ANOVA was performed in **C**, Student’s *t*-test was performed in **B**, **F**, **G**, **H, J, L** and **M**. D indicates day; W, week; CBB, Coomassie Brilliant Blue staining; TAC, transverse aortic constriction. NT-proBNP, N-Terminal Pro-B-Type Natriuretic Peptide. HCS, healthy control subjects; HrCD, hypertension-related cardiac dysfunction.

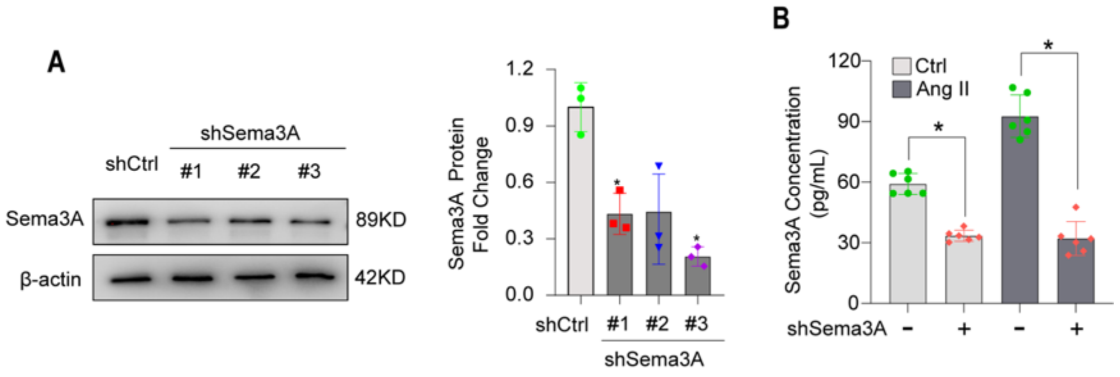

Supplemental Figure S4. Knock-down of Sema3A in MiVECs by short hairpin RNA small (shRNA).

A, Immunoblotting analysis of Sema3A protein expression in MiVECs transfected with empty shRNA (shCtrl) lentivirus or Sema3A shRNAs #1-#3 lentivirus; n=3 samples per group. **B-C**, Sema3A secreted into the culture medium was measured by ELISA (n = 6 samples per group). All data are presented as mean ± SEM, * *P* < 0.05, One-way ANOVA was performed in **A**, Student's *t*-test was performed in **B** and **C**.

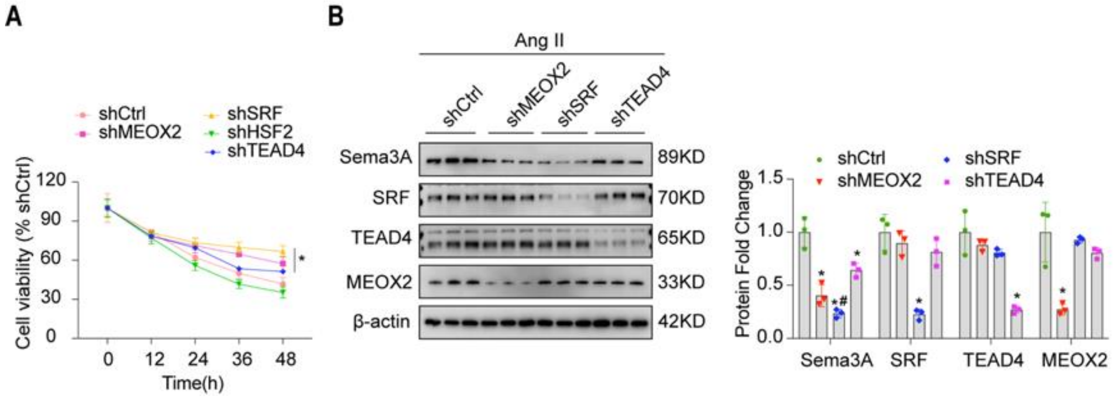

Supplemental Figure S5. Effects of knockdown of potential transcription factors on cell viability and Sema3A expression in MiVECs under Ang II treatment.

A, Cell viability was analyzed by CCK-8 assay in MiVECs with knockdown of potential transcription factors (MEOX2, SRF, HSF2, and TEAD4) under Ang II treatment. n = 3 samples per group. **P* < 0.05 by one-way ANOVA. **B,** Expression of Sema3A in MiVECs with knockdown of MEOX2, SRF, and TEAD4 was detected by immunoblotting assay. n = 3 samples per group. **P* < 0.05 by one-way ANOVA.

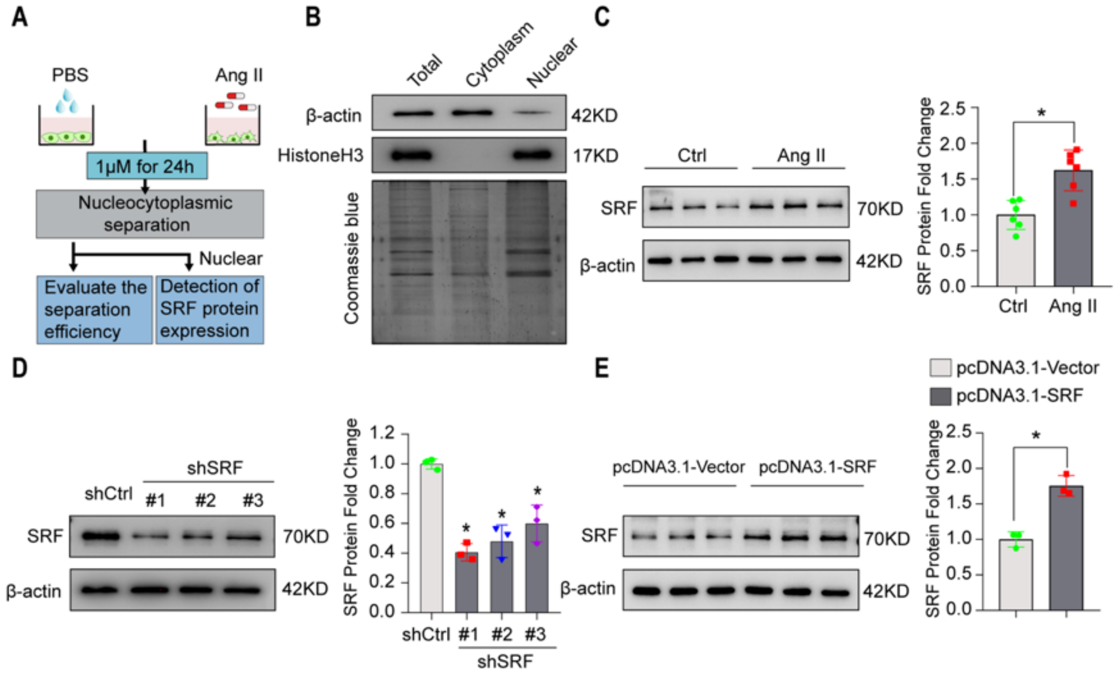

Supplemental Figure S6. Immunoblotting analysis of the expression of SRF protein.

A, Illustration of the experimental design for nucleocytoplasmic cell separation. **B** MiVECs were fractionated to separate the nucleus and cytoplasm. β-actin and histone H3 were used as positive controls in the cytoplasm and nucleus, respectively. Total protein levels were determined using blots stained with Coomassie Brilliant Blue. **C**, Immunoblotting analysis of SRF proteins in the normal control and Ang II-infused mouse hearts. β-actin was used as the loading control. n = 6 mice per group. **D**, Immunoblotting analysis of the expression of SRF protein in MiVECs transduced with empty shRNA (shCtrl) lentivirus or SRF shRNAs#1–#3 lentivirus; n = 3 samples per group. **E** SRF protein expression was detected by immunoblotting. All statistical data are presented as mean ± SEM; **P* < 0.05; Student's *t*-test was performed in **C** and **E**, and one-way analysis of variance (ANOVA) was performed in **D**.

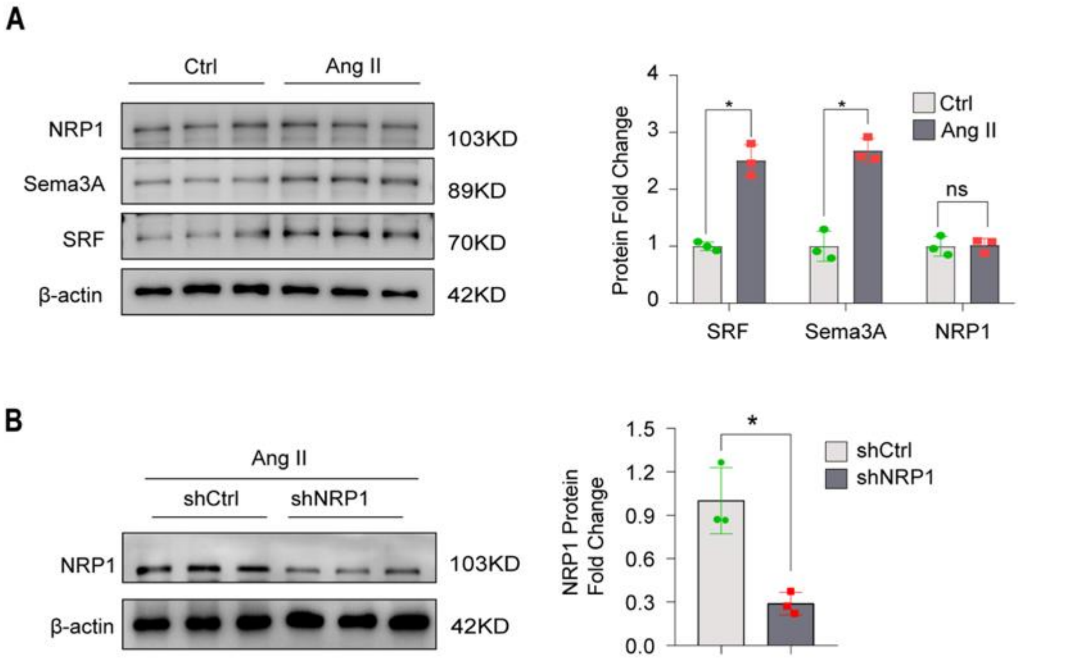

Supplemental Figure S7. Expression of NRP1, Sema3A, and SRF detected by Immunoblotting analysis.

A, Representative immunoblot images (left panel) and quantitative analysis (right panel) of NRP1, Sema3A, and SRF levels with or without Ang II treatment. $*P < 0.05$, compared to the control group; n = 3 samples per group. **B**, NRP1 protein expression in MiVECs transfected with either a control shRNA lentivirus (shCtrl) or shRNA targeting NRP1 (shNRP1) by immunoblotting. n = 3 samples per group. For all statistical plots, the data are presented as mean ± SEM and were analyzed by Student's *t*-test. $*P < 0.05$. ns indicates no significant difference.

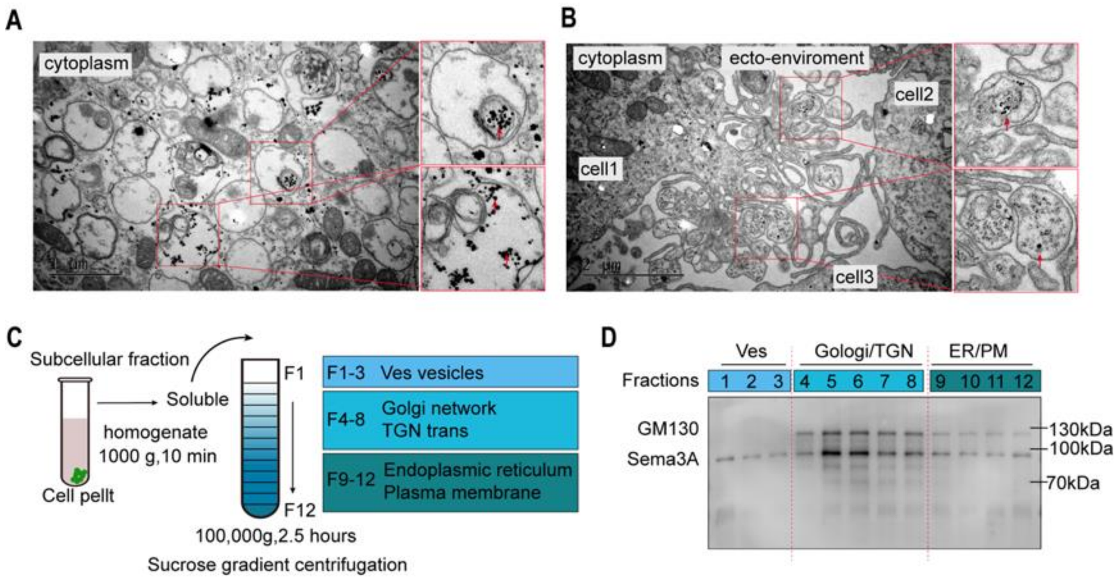

Supplemental Figure S8. Localization of Sema3A proteins in MiVECs

A-B, Immunoelectron microscopy studies of Sema3A in MiVECs. Ultrathin sections were examined using transmission electron microscopy. High-density colloidal gold particles were indicative of Sema3A. **A**, Arrows indicate Sema3A expression in multivesicular body (MVB)-like vesicles. Scale bar = 1 μm. **B**, A vesicle containing Sema3A protein appears ready to undergo exocytosis. Scale bar = 2 μm. **C** Schematic overview of subcellular fractionation and sucrose density gradient centrifugation. **D**, Immunoblotting analysis of the subcellular localization of Sema3A in MiVECs using sucrose density gradient fractionation. Ves indicates vesicles; ER, endoplasmic reticulum; PM, plasma membrane; TGN, trans-Golgi network.

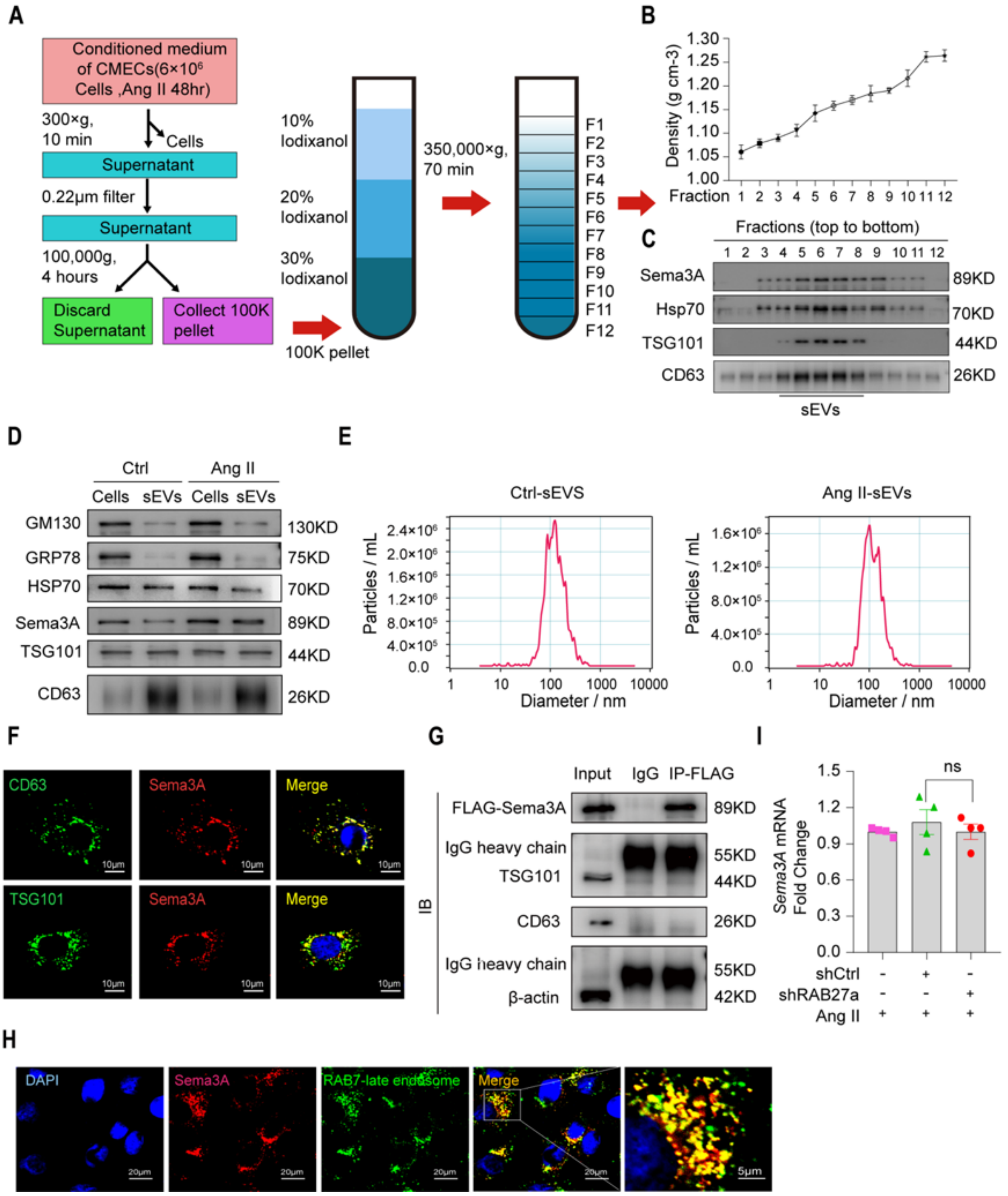

Supplemental Figure S9. Extraction, identification, and localization of small extracellular vesicles.

A, Experimental workflow of cushioned-density gradient ultracentrifugation (C-DGUC) for sEVs isolation. **B**, Density analysis of iodixanol gradient fractions (g/ml). **C**, Immunoblotting analysis of sEV-specific markers and Sema3A abundance in subfractions after iodixanol gradient flotation. **D**, Immunoblotting analysis of the protein composition of sEVs isolated from MiVECs treated with PBS or Ang II. **E,** Measurement of sEV concentration using nanoparticle tracking analysis (NTA). **F**, Sema3A colocalization with CD63 and TSG101 in MiVECs. Sema3A was stained red,

CD63 and TSG101 were stained green, and nuclei were counterstained with DAPI. Scale bar = 10 μm. **G**, Immunoprecipitation assay using anti-FLAG antibody in cell lysates from MiVECs transduced with a plasmid expressing FLAG-Sema3A. **H**, Representative confocal microscopy images of MiVECs assessing the colocalization of biotin-labeled Sema3A and late endosomes (denoted by RAB7). Scale bar = 20 μm. **I**, qRT-PCR quantification of *Sema3A* expression; n = 3 samples per group; data are presented as mean ± SEM, $*P < 0.05$, one-way analysis of variance (ANOVA). IP, immunoprecipitation; IB, Immunoblotting; ns, not significant.

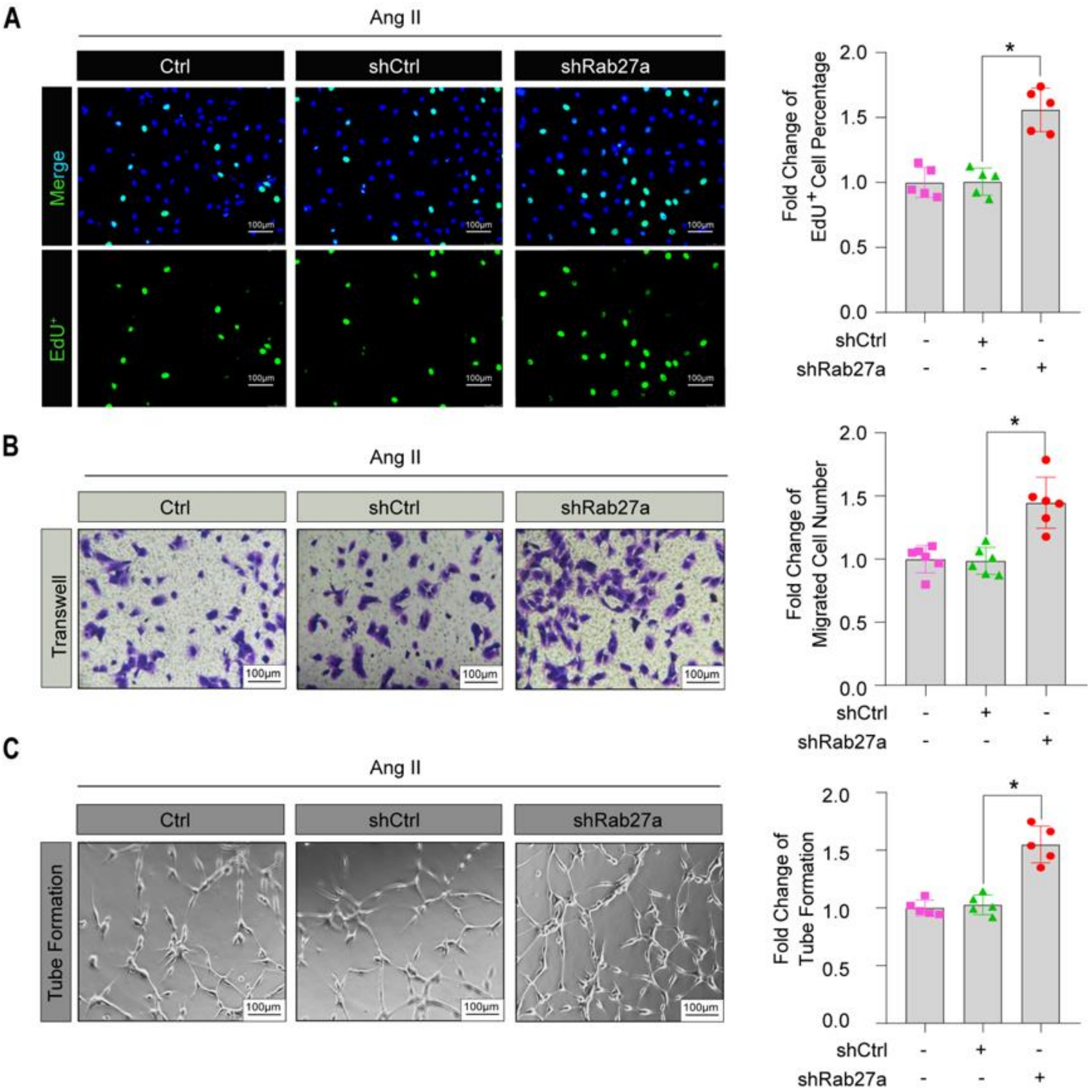

Supplemental Figure S10. Silencing Rab27a suppresses sEV release and mitigates Ang II-induced angiogenic neovascularization impairment.

A Representative EdU images (left panel, scale bar = 100 μm) and quantification of

MiVEC proliferation (right panel). **B**, Representative Transwell assay images (left panel, scale bar = 100 μm) and quantification of MiVEC migration (right panel). **C** Representative images of MiVECs in the tube formation experiment. Scale bar = 100 μm. All statistical data are presented as mean ± SEM; **P* < 0.05, one-way analysis of variance (ANOVA). EdU, 5-ethynyl-2′-deoxyuridine.

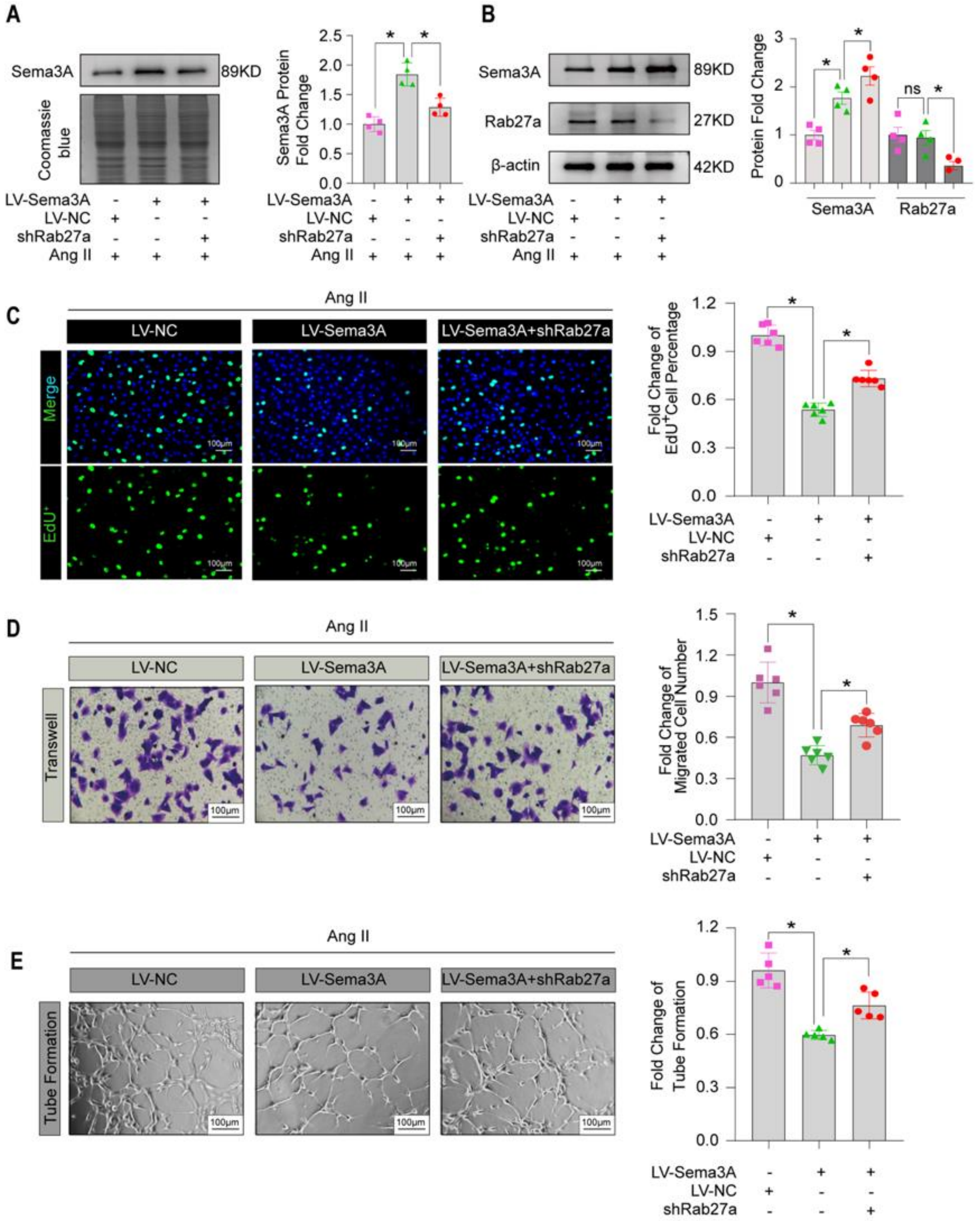

Supplemental Figure S11. Blocking the release of extracellular vesicles attenuated Ang II-induced dysfunction of MiVECs.

A, Immunoblotting analysis of Sema3A protein levels in the culture medium. Coomassie brilliant blue (staining shows the protein loading amount). n = 4 samples per group. **B**, Whole cell lysates of MiVECs were analyzed by immunoblotting for Sema3A or Rab27 expression. β-actin was used as the loading control. n=4 samples per group. **C-E**, Representative EdU (**C**), Transwell (**D**), and tube formation (**E**) images show the effect of Sema3A on MiVEC proliferation, migration, and tube formation after inhibiting sEV secretion by *Rab27a* knockdown. Scale bar = 100 μm; n = 5 samples per group. All statistical data are presented as mean ± SEM; * $P < 0.05$, one-way analysis of variance (ANOVA). ns indicates no significant difference.

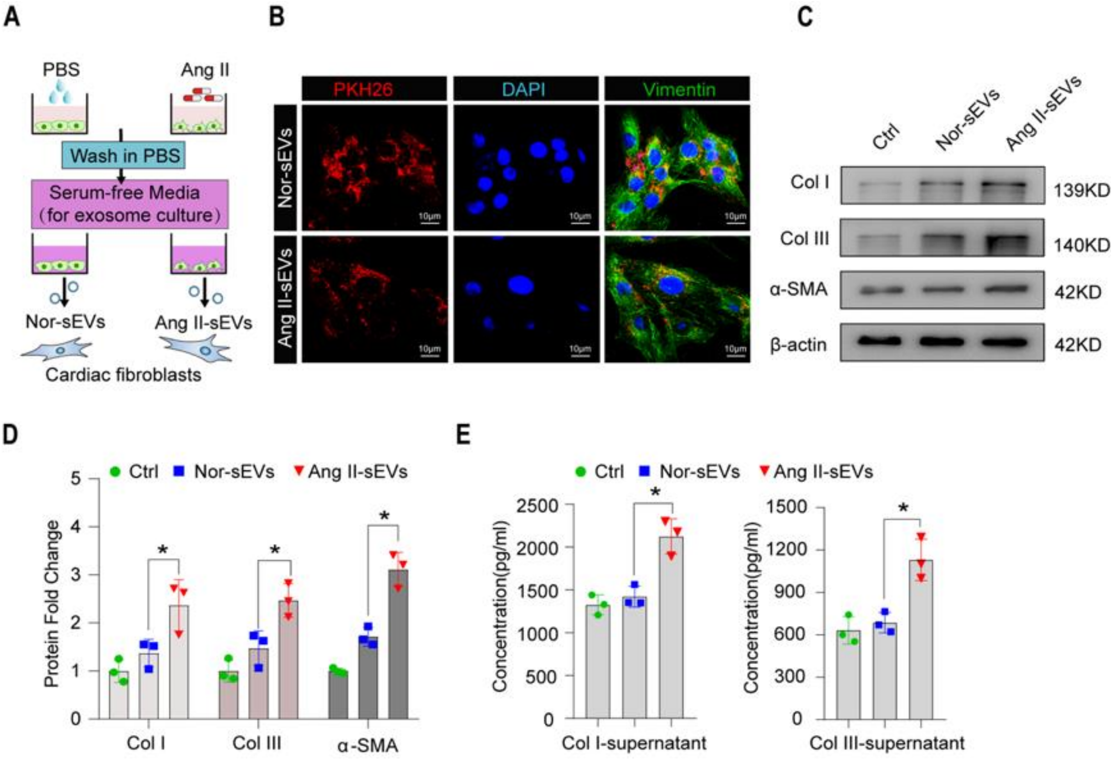

Supplemental Figure S12. MiVEC-derived Sema3A-containing small extracellular vesicle activated fibroblast activation.

A, Schematic illustration of the experimental approaches. **B**, Cellular uptake of PKH26 (red)-labelled sEVs. sEVs from MiVECs were labeled with PKH26 and added to cardiac fibroblast cultures. Scale bar = 10 μm. **C-D**, Immunoblotting analysis of collagen-related protein (Collagen I, Collagen III, and α-SMA) expression in cardiac fibroblasts treated with Nor-sEVs or Ang II-sEVs. n = 3 samples per group. **E**, ELISA analysis of Col I and Col III secreted from cardiac fibroblasts after Nor-sEV or Ang II-sEVs treatment. n = 3 samples per group. All statistical data are presented as mean

± SEM; * $P < 0.05$, one-way analysis of variance (ANOVA). Col I, Collagen type1; Col III, Collagen type3.

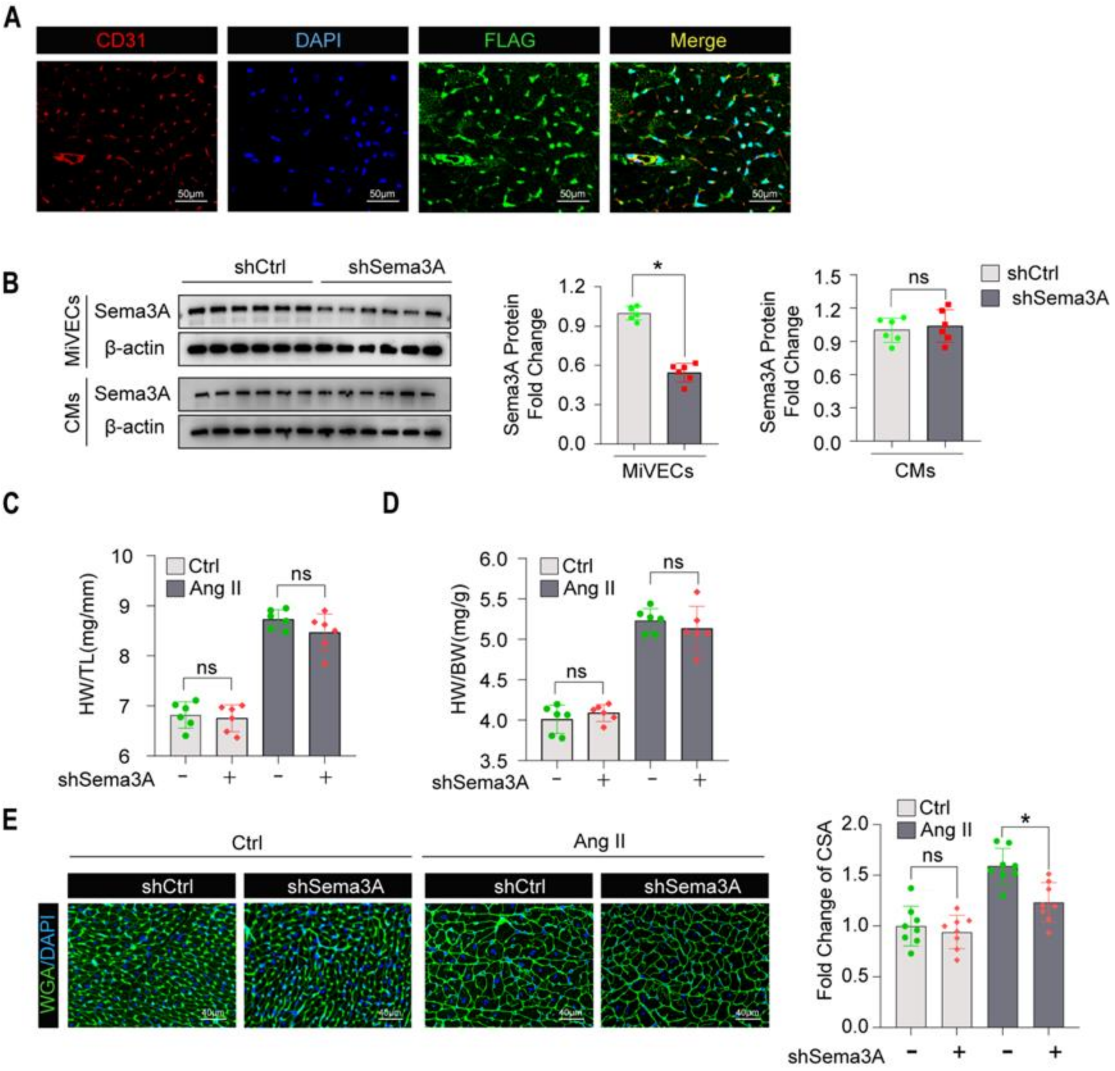

Supplemental Figure S13. Knockdown efficiency of Sema3A in cardiac microcirculation.

A, Immunofluorescence staining of FLAG (green) and CD31 (red) in AAV9-ENT-ICAM2-infected hearts. Scale bar = 50 μm. **B** Immunoblotting was performed to quantify Sema3A expression in MiVECs and cardiomyocytes isolated from AAV9-ENT-ICAM2-control (shCtrl) or AAV9-ENT-ICAM2-shSema3A (shSema3A)-infected hearts; n = 6 mice per group. **C**, Heart weight/body weight (HW/BW) and **D**, heart weight/tibia length (HW/TL) ratios. n = 6 mice per group. **E**, Heart sections were stained with WGA-FITC (green) to demarcate cell boundaries. Scale bar = 40 μm. n = 6 mice per group. All statistical data are presented as mean ± SEM; *$P < 0.05$; **B**, **C**, **D** and **E** were analyzed using Student's *t*-test. CMs, cardiomyocytes;

DAPI, 4′,6-diamidino-2-phenylindole; ns, not significant.

Supplemental Table 4. Clinical and laboratory characteristics of the study participants.

Groups	General population	HFpEF	*P* value
Age (years)	56.56 ± 13.70	61.73 ± 12.73	0.1028
Sex, (male/female)	32 (18/14)	40 (23/17)	_
BMI (kg/m^2)	23.14 ± 3.592	21.65 ± 3.264	0.0704
SBP (mmHg)	127.40 ± 18.10	123.90 ± 20.97	0.4502
DBP (mmHg)	77.34 ± 12.46	54.78 ± 7.59	0.5959
LVEF (%)	62.44 ± 6.61	56.75 ± 5.60	0.0002
cTnT (μg/mL)	39.75 ± 60.99	42.25 ± 57.64	0.8591
NT-proBNP (pg/mL)	320.2 (172.5-472.8)	2156.0 (1450.0-3143.0)	<0.0001
Sema3A (ng/mL)	62.48 ± 29.54	62.48 ± 29.54	<0.0001

Data are presented as mean ± SD for normally distributed values or median (25-75th percentiles) for nonparametric values. GP, general population; HFpEF, heart failure with preserved ejection fraction; BMI, body mass index; SBP, systolic blood pressure; DBP, diastolic blood pressure; NT-proBNP, N-Terminal Pro-B-Type Natriuretic Peptide.

SUPPLEMENTAL REFERENCES

[1] T. Wilhelmi, X. Xu, X. Tan, M. S. Hulshoff, S. Maamari, S. Sossalla, M. Zeisberg, E. M. Zeisberg, *Theranostics* **2020**, *10* 3905 DOI: 10.7150/thno.38640.

[2] M. Ackers-Johnson, P. Y. Li, A. P. Holmes, S. M. O'Brien, D. Pavlovic, R. S. Foo, *Circ. Res.* **2016**, *119* 909 DOI: 10.1161/CIRCRESAHA.116.309202.

[3] P. Gutwein, A. Stoeck, S. Riedle, D. Gast, S. Runz, T. P. Condon, A. Marmé, M. C. Phong, O. Linderkamp, A. Skorokhod, P. Altevogt, *Clin. Cancer Res.*

2005, *11* 2492 DOI: 10.1158/1078-0432.CCR-04-1688.

[4] J. Kowal, G. Arras, M. Colombo, M. Jouve, A CTJPNASUS, *Proc. Natl Acad. Sci. U. S. A.* **2016**, *113* 968 DOI: 10.1073/pnas.1521230113.

[5] C. Théry, S. Amigorena, G. Raposo, A. J. C. ClaytonPiCB, *Curr. Protoc. Cell Biol.* **2006**, *13* 12 DOI: 10.1002/0471143030.cb0322s30.

[6] Y. Tian, M. Gong, Y. Hu, H. Liu, W. Zhang, M. Zhang, X. Hu, D. Aubert, S. Zhu, L. Wu, *J. Extracell. Vesicles* **2019**, *9* 1697028 DOI: 10.1002/0471143030.cb0322s30.

[7] H. Liu, Y. Tian, C. Xue, Q. Niu, C. Chen, Yan XJJoev, *J. Extracell. Vesicles* **2022**, *11* 12206 DOI: 10.1002/jev2.12206.

[8] M. Inoue, Y. Wakayama, M. Murahashi, S. Shibuya, T. Jimi, H. Kojima, H. Oniki, *Acta Neuropathol.* **1996**, *92* 569 DOI: 10.1007/s004010050563.

[9] I. Guillemin, M. Becker, K. Ociepka, E. Friauf, H. G. Nothwang, *Proteomics* **2005**, *5* 35 DOI: 10.1002/pmic.200400892.

[10] P. Gutwein, A. Stoeck, S. Riedle, D. Gast, S. Runz, T. P. Condon, A. Marmé, M. C. Phong, O. Linderkamp, A. Skorokhod, P. Altevogt, *Clin. Cancer Res.* **2005**, *11* 2492 DOI: 10.1158/1078-0432.CCR-04-1688.

[11] S. Yoon, G. H. Eom, *Exp. Mol. Med.* **2019**, *51* 1 DOI: 10.1038/s12276-019-0323-2.

[12] K. Varadi, S. Michelfelder, T. Korff, M. Hecker, M. Trepel, H. A. Katus, J. A. Kleinschmidt, O. J. Müller, *Gene Ther.* **2012**, *19* 800 DOI: 10.1038/gt.2011.143.
